# Integrated Personal Health Record in Indonesia: Design Science Research Study

**DOI:** 10.2196/44784

**Published:** 2023-03-14

**Authors:** Nabila Clydea Harahap, Putu Wuri Handayani, Achmad Nizar Hidayanto

**Affiliations:** 1 Faculty of Computer Science University of Indonesia Depok Indonesia

**Keywords:** personal health record, integrated, Indonesia, design science, mobile phone

## Abstract

**Background:**

Personal health records (PHRs) are consumer-centric tools designed to facilitate the tracking, management, and sharing of personal health information. PHR research has mainly been conducted in high-income countries rather than in low- and middle-income countries. Moreover, previous studies that proposed PHR design in low- and middle-income countries did not describe integration with other systems, or there was no stakeholder involvement in exploring PHR requirements.

**Objective:**

This study developed an integrated PHR architecture and prototype in Indonesia using design science research. We conducted the research in Indonesia, a low- to middle-income country with the largest population in Southeast Asia and a tiered health system.

**Methods:**

This study followed the design science research guidelines. The requirements were identified through interviews with 37 respondents from health organizations and a questionnaire with 1012 patients. Afterward, the proposed architecture and prototype were evaluated via interviews with 6 IT or eHealth experts.

**Results:**

The architecture design refers to The Open Group Architecture Framework version 9.2 and comprises 5 components: architecture vision, business architecture, application architecture, data architecture, and technology architecture. We developed a high-fidelity prototype for patients and physicians. In the evaluation, improvements were made to add the stakeholders and the required functionality to the PHR and add the necessary information to the functions that were developed in the prototype.

**Conclusions:**

We used design science to illustrate PHR integration in Indonesia, which involves related stakeholders in requirement gathering and evaluation. We developed architecture and application prototypes based on health systems in Indonesia, which comprise routine health services, including disease treatment and health examinations, as well as promotive and preventive health efforts.

## Introduction

### Background

Current trends in health informatics encourage the transition from institution-centric to patient-centric health care [1]. The use of IT is not only for patients in health care settings but also for all individuals who want to maintain health and are involved in disease prevention and health promotion [1]. Personal health records (PHRs) are consumer-centric tools designed to facilitate the tracking, management, and sharing of personal health information [1]. PHRs contain medical data and information about a patient that are managed by the patients themselves [2]. PHRs form a trend from information controlled by the health system to information controlled by individuals [3].

In its simplest form, a PHR is a stand-alone application (stand-alone PHR) and is not connected to other systems [4]. In a more complex form, the health information provided by the PHR is linked to the electronic health record or electronic medical record (tethered PHR) [4]. Furthermore, PHRs can be connected to various health data sources to obtain and transmit data (integrated PHR) [4]. An integrated PHR is the most ideal form of PHR as implementing PHRs in this way has the potential to improve the quality, accessibility, and delivery of health services [3].

A previous review on the implementation of PHRs shows that PHR research has mainly been conducted in high-income countries rather than in low- and middle-income countries [5]. Few studies that have been conducted in low- and middle-income countries aim to propose PHR applications for certain purposes, such as pediatric vaccination [6], or specific diseases, such as metabolic syndrome management [7], chronic heart failure [8], and kidney transplant [9]. These studies focused on the usability of PHRs and did not describe integration with other systems or applications. A study by Abdulnabi et al [10] described PHR interoperability by designing a distributed PHR model. However, there was no stakeholder involvement in exploring PHR requirements.

Using design science, we complement gaps from previous studies by developing a PHR model that is integrated with various systems, and we involve relevant stakeholders to explore the requirements and evaluate the proposed PHR model. We conducted the research in Indonesia, a low- to middle-income country with the largest population in Southeast Asia [11,12]. In Indonesia, health services are delivered by the public and private sectors. In the public sector, health facilities comprise hospitals (general and specialty) and *pusat kesehatan masyarakat* or primary health centers (Puskesmas). In the private sector, health facilities comprise hospitals and primary care clinics. In addition, there is the Social Security Agency for Health or *Badan Pelaksana Jaminan Sosial Kesehatan* (BPJS Kesehatan) that administers the national health insurance program (*Jaminan Kesehatan Nasional* [JKN] or national health insurance). Patients with JKN must follow a tiered referral flow starting from primary care facilities as gatekeepers for JKN patients before being referred to hospitals. Without a referral letter, JKN patients are not allowed to go directly to a hospital or specialist clinic except in an emergency [13].

In addition to health efforts that focus on treating and curing diseases, there are also Healthy Family (Keluarga Sehat) and Community Healthy Life Movement (Germas) programs that are managed by the Puskesmas and focus on promotive and preventive health efforts [14,15]. Currently, health development policies in Indonesia are directed at improving access to and quality of health services, with an emphasis on increasing promotive and preventive health efforts supported by innovation and the use of technology [16]. Integrated PHRs can be an opportunity to improve access to and quality of health services in Indonesia by using IT [17].

### Objectives

As a technological solution for integrated PHRs in Indonesia, this study developed an integrated PHR architecture and prototype in Indonesia using the design science research (DSR) approach by Hevner et al [18]. The DSR approach was chosen in this study as the goal of DSR is to focus on designing systems that not only are practical but can also contribute to knowledge. The question that will be answered in this research is as follows: How are the architectural designs and prototypes of integrated PHR applications in Indonesia? The design of the PHR model, which was developed using a DSR approach, can provide an overview for developing PHRs using scientific theory and methods. The results of this study are expected to be a guide for health facilities or health policy makers in integrating PHRs and health applications in Indonesia.

## Methods

### Design Science

**Figure 1:**
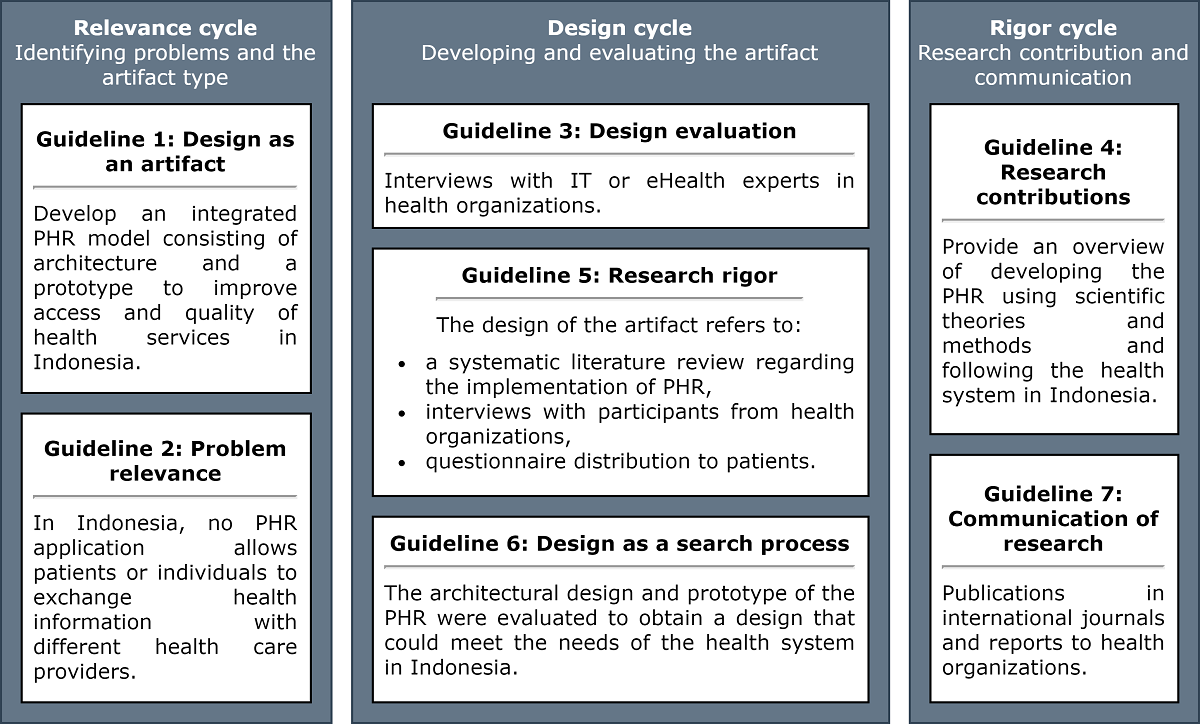
Design science research guideline [5,18]. PHR: personal health record.

This study was conducted using a DSR approach [18]. In DSR, the details or stages of design and development may vary even though the focus of the research is on artifact design [19]. Peffers et al [20] describe 6 activity-based methodologies for DSR, whereas Hevner et al [18] define 7 guidelines for DSR. This study follows the DSR guidelines defined by Hevner et al [18], which are grouped into 3 cycles or phases comprising the relevance cycle (identifying problems and the artifact type), design cycle (developing and evaluating the artifact), and rigor cycle (research contribution and communication; Figure 1 [5]). These guidelines have been followed by DSR studies in low- and middle-income countries to develop health information systems such as mobile health (mHealth) [19,21-27]. The guidelines by Hevner et al [18], as a necessary element in DSR, are also consistent with the DSR methodology by Peffers et al [20].

The artifacts developed in this study are architecture and application prototypes. Integrated PHR requirements were carried out based on a systematic literature review regarding functionalities and issues in the implementation of PHRs [5], literature studies on health regulations in Indonesia, interviews with health organizations, and questionnaire distribution to patients. The requirements mentioned by health organizations in the interviews were categorized and grouped into themes that were defined by Harahap et al [5] and combined with requirements from patients. The architectural design then became a reference for designing the application prototype.

This study used a purposive sampling method to select participants who had the required knowledge regarding PHR implementation. The health organizations from which participants were interviewed were health facilities (private and government hospitals, Puskesmas, and clinics), health regulators (Ministry of Health and BPJS Kesehatan), and health application vendors. Data were collected through semistructured interviews between August 19, 2020, and January 15, 2021, and between October 1, 2021, and October 11, 2021. Interviews were conducted on the web using Zoom Cloud Meetings (Zoom Video Communications) and audio recorded with the participants’ consent. Each interview lasted between 30 and 60 minutes. The interview questions are attached in Multimedia Appendix 1.

The questionnaire was distributed to patients who met the criteria of respondents being Indonesian citizens aged ≥17 years. Before the questionnaire was distributed, we conducted a readability test to ensure that all the items on the questionnaire could be understood by the respondents in terms of writing and sentence meaning. The readability test was conducted on 6 respondents from September 25, 2021, to September 28, 2021. After the readability test, we made a revision based on the input given by the respondents. Questionnaires were distributed on the web through messaging applications such as WhatsApp (Meta Platforms), Telegram (Telegram FZ LLC), and Line (Line Corporation), as well as social media such as Facebook and Twitter, from October 28, 2021, to November 20, 2021. Questionnaire data were analyzed using descriptive statistics in Microsoft Excel (Microsoft Corp) and SPSS Statistics (version 28; IBM Corp).

To evaluate the artifacts, this study used the evaluation guidelines defined by Venable et al [28] and the evaluation criteria defined by Hevner et al [18]. The goal of the evaluation was to determine the suitability of the design for the needs of health services in Indonesia. The architectural design and application prototype were evaluated qualitatively through interviews with IT or eHealth experts. The evaluation of the architecture design aimed to assess the completeness and conformity with the health system in Indonesia. The evaluation of the application aimed to assess functionality and usability.

This study used the COREQ (Consolidated Criteria for Reporting Qualitative Research) guidelines as a comprehensive checklist that covers the necessary components of qualitative research (Multimedia Appendix 2 [29]). Interview data were analyzed using content analysis techniques in NVivo (version 12; QSR International). The content analysis steps comprised decontextualization, recontextualization, categorization, and compilation [30]. In decontextualization, the authors read the transcribed text and broke down the text into smaller meaning units. Each identified meaning unit was labeled with a code. In recontextualization, the original text was reread alongside the final list of meaning units. In the categorization process, themes and categories were identified. The categorization for the requirement analysis was based on the functionalities and issues in the implementation of PHRs defined by Harahap et al [5]. The categorization for the architectural evaluation interview was carried out based on the architectural components, whereas the categorization for the prototype evaluation interview was carried out based on the implemented functions in the PHR application. At the compilation stage, the authors wrote the results of the analysis.

### Ethics Approval

The authors obtained a letter of ethics approval from the Faculty of Computer Science, University of Indonesia, to conduct data collection with letter S-1122A/UN2.F11.D1/PDP.01/2020. The author submitted the letter to the respondents and provided a brief explanation of the study objective. Each respondent verbally provided consent to participate during the interview.

## Results

### Artifact Development

#### Respondent Demographics

We interviewed a total of 37 respondents. The respondents were from 10 first-level health facilities (n=6, 60% Puskesmas and n=4, 40% clinics) and 15 referral-level health facilities (n=9, 60% government hospitals and n=6, 40% private hospitals); 5% (2/37) of respondents were from the Ministry of Health, 3% (1/37) of respondents were from BPJS Kesehatan, and 8% (3/37) of respondents were health application vendors. A total of 73% (27/37) of respondents were male, and 27% (10/37) were female. Most respondents (24/37, 65%) were from the Special Capital Region of Jakarta, whereas others were from Jawa Barat (6/37, 16%), Bali (3/37, 8%), Banten (1/37, 3%), the Special Region of Yogyakarta (1/37, 3%), Riau (1/37, 3%), and Sulawesi Selatan (1/37, 3%). Detailed respondent information is provided in Multimedia Appendix 3.

A total of 1343 respondents filled out the questionnaire. However, there were 24.65% (331/1343) of invalid or duplicate data, so the total valid data from filling out the questionnaire were from 75.35% (1012/1343) of respondents. A total of 37.55% (380/1012) of respondents were male, 62.45% (632/1012) were female, and most (606/1012, 59.88%) lived in Greater Jakarta. Most respondents were aged 20 to 30 years (376/1012, 37.15%; Table 1).

**Table 1:** Demographics of questionnaire respondents (n=1012).

Demographics		Respondents, n (%)
**Sex**		
	Male	380 (37.5)
	Female	632 (62.5)
**Age (years)**		
	17-20	207 (20.5)
	20-30	376 (37.2)
	31-40	148 (14.6)
	41-50	134 (13.2)
	51-60	125 (12.4)
	>60	22 (2.2)
**Domicile**		
	Greater Jakarta	606 (59.9)
	Java island other than Greater Jakarta	279 (27.6)
	Outside Java island	78 (7.7)
**Education level**		
	Primary school	0 (0)
	Junior high school	1 (0.1)
	Senior high school	315 (31.1)
	Diploma	62 (6.1)
	Bachelor’s degree	415 (41)
	Master’s degree	173 (17.1)
	Doctorate	46 (4.5)
**Familiarity with the use of IT**		
	Excellent	298 (29.4)
	Good	519 (51.3)
	Okay	191 (18.9)
	Bad	2 (0.2)
	Very bad	2 (0.2)

#### Requirements

##### Health Organization Requirements

The functions recommended by respondents from health organizations included functions related to access to health records for patients, such as viewing diagnostic data, laboratory and examination results, and medical history:

They can view data from medical visits, such as lab results, and x-ray results. Then, you can also see the medical history, including the diagnosis.

Head of IT, General hospital (GH) 3

Respondents also mentioned the need for a function to view health facility profiles, such as the list of services and the availability of beds. Other recommended functions were paying for medical expenses, billing, and claiming health insurance:

List of services that can be provided because each hospital is different.

Physician, GH8

If there is a bill for the patient, it will be created to be given to the patient.

Health application vendor, vendor (VDR) 1

Respondents also suggested the need for a function for patients to manage information related to medication consumption, such as viewing the history of medication that has been consumed, ordering medication, and scheduling medication consumption:

Ordering medicine according to a prescription, then delivery of the medicine.

Health application vendor, VDR3

There is a function for medication consumption.

Head of IT, primary health care (PHC) 8

Another recommended function was a feature that patients can use to interact or communicate on the web with medical personnel in health facilities. Communication can occur through chat, messages related to health consultations, or video calls:

Communication to hospital and telemedicine with video.

Head of IT, private hospital (PH) 3

In addition, respondents mentioned the need for features for patients to manage appointments with medical personnel at health facilities. With this feature, patients can register themselves for treatment at health facilities, including choosing a physician:

There is an online registration for patients...there must be information on what time they should be treated.

Head of IT, GH6

Respondents also recommended a function for patients to access disease-related information and health tips, such as how to maintain a healthy lifestyle. Others suggested a function for patients to manage their health data to support preventive health efforts or disease prevention. In addition, this function could assist in the recovery from certain diseases that require ongoing health management activities. For example, patients could input data on vital signs and physical activities:

There are health articles that can be used when other features are not used. The available information depends on patients’ health conditions. This could include articles on healthy lifestyles such as safe cosmetics, or nail care.

Head of IT planning strategy, health regulator (HR) 2

Monitoring tracking is also a very good opportunity because health is not only about curing, we also need preventive measures so that we don’t get sick.

Head of IT, PH2

In addition to functional requirements, respondents mentioned the need for PHR integration. PHR functionality can be integrated with health applications or other existing data sources, such as electronic medical records in health facilities, to obtain medical summaries, such as diagnoses, laboratory and examination results, and medical history. PHRs also need to be integrated with the referral information system (*sistem informasi rujukan terintegrasi*) to obtain patient referral history and integration with vaccine data, especially for needs during the COVID-19 pandemic:

It needs to be integrated with electronic medical record data from health facilities.

Member of data and information center, HR1

We need to integrate data from SISRUTE because it records data from several health facilities. If the patient is referred, the data should be recorded.

Head of IT, GH5

PHRs also need to be integrated with BPJS Kesehatan for JKN patients and integrated with web-based payments for patients who seek treatment at health facilities and do not use health insurance:

Need to be integrated with BPJS health.

Head of IT, GH3

For payments, it is integrated with online payments.

Health application vendor, VDR3

PHRs can be integrated with teleconsultation applications for communication between patients and medical personnel, as well as with pharmacies for ease of ordering medicines. In addition, for the convenience of monitoring personal health data such as physical activities, PHRs need to be integrated with wearable devices. Moreover, PHRs need to be integrated with the healthy family or *Program* Indonesia *Sehat dengan Pendekatan Keluarga* application to support healthy family programs at the Puskesmas and with the national health data repository owned by the Ministry of Health:

For prescription, the application needs to be connected to the pharmacy.

Physician, PH5

Regarding fitness, it is difficult to implement, unless you can access data from wearable devices such as smartwatches.

Member of data and information center, HR1

We have a healthy family application (PIS-PK) to record family information related to individual family members, and it is not from the results of the medical examination.

Head of IT, HR1

Respondents also mentioned security aspects that need to be applied to PHRs, such as access control, audit trails, data encryption, and data backup. Authentication and authorization are needed in the implementation of PHRs. An audit trail is required to review who is accessing and what data have been accessed in the PHR. A data backup option is required to avoid the risk of data loss. In addition, PHRs need to implement important data encryption, such as passwords:

There must be a data backup.

Head of IT, GH2

There is a log in the application to see what time the user logged in and what features were accessed.

Head of IT, PHC1

PHRs need to implement user manuals or guidance options to help users understand the information contained in them. In addition, PHRs need to have customization options based on the availability of the internet network as several regions in Indonesia have poor internet connections:

Provide user manuals, video manuals, or readable manuals.

Health application vendor, VDR1

For areas with poor internet network, the application should still be accessible.

Physician, GH8

##### Patient Requirements

A total of 70.06% (709/1012) of respondents had used health applications, whereas 29.94% (303/1012) had never used health applications. For each respondent who had used a health application, the questions asked were the health application used, the platform used to access the health application, the length of use of the health application, the frequency of use of the health application in the last 6 months, the features used in the health application, reasons for using health applications, challenges when using health applications, organizations that must be integrated or connected with health applications, and the most important components of a health application (Multimedia Appendix 4).

**Figure 2:**
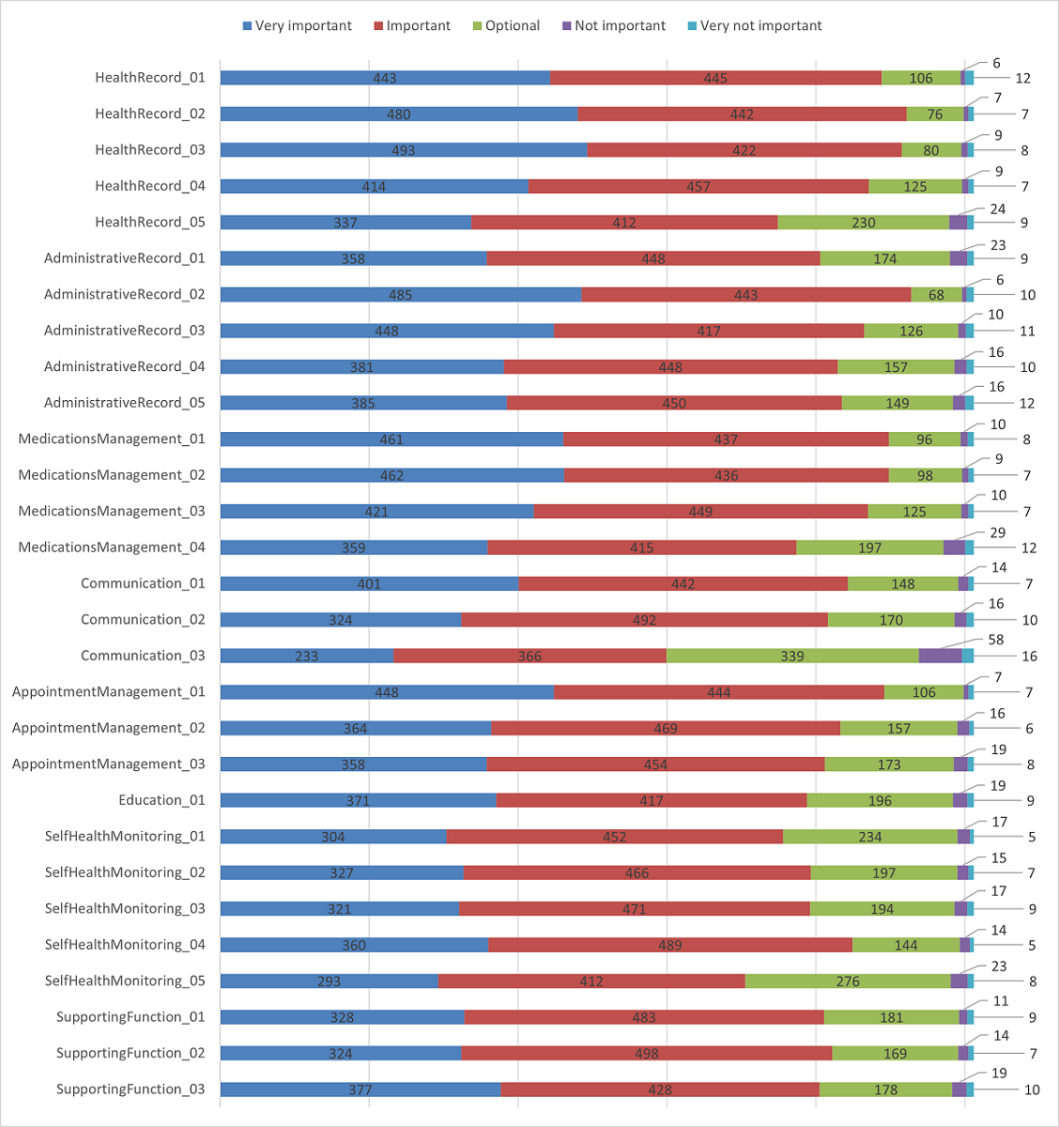
Respondents’ scores for each personal health record functionality.

Respondents were also asked to rate how important the PHR functionalities were based on the previous review (Harahap et al [5]), which comprised health records, administrative records, medication management, communication, appointment management, education, self–health monitoring, and supporting function. The functionality codes for each PHR module are summarized in Multimedia Appendix 5. Respondents were asked to provide an assessment with the following scores: 1=*Very Not Important*, 2=*Not Important*, 3=*Optional*, 4=*Important*, and 5=*Very Important*. The mean was then calculated for each functionality. If the mean of the functionality was <4, then the functionality did not need to be implemented in the integrated PHR model. On the basis of the results of the questionnaire, 27 functionalities had a mean of >4. However, the functionality of sending messages or chatting with support groups and family members in the PHR application (Communication_03) and the functionality to connect wearable devices with the PHR application were considered important by fewer respondents (SelfHealthMonitoring_05), with a mean of <4. Figure 2 shows the respondents’ scores for each PHR functionality.

##### Summary of User Requirements

The requirements of health organizations and patients were grouped into PHR modules and functionalities based on the study by Harahap et al [5], which comprised health records, administrative records, medication management, communication, appointment management, education, and self–health monitoring. In addition, there were emergency modules obtained from patients’ requirements as well as security modules and supporting functions to meet the nonfunctional requirements of PHRs. In the health record module, there were functions to view the results of medical examinations, medical history, referrals, and vaccinations. In the administrative records module, there were functions for patient profiles, health facility profiles, physician profiles, health insurance, and payments and billing. In the medication management module, there were functions for medication history, medication reminders, and medication orders. In the communication module, there was a messaging function (SMS text message or video call). In the appointment management module, there were functions for registration, appointment history, reminders or notifications, and ambulance. In the education module, there was a health article function. In the self–health monitoring module, there were health data tracking functions, health dashboards, health calculators, and early warning notifications. In the emergency module, there was an emergency contact function. In the security module, there were authentication, authorization, audit log, and backup functions. In the supporting function module, there were user manual and offline functionalities. A summary of the PHR module and functionality based on the requirements of the respondent group is provided in Multimedia Appendix 6.

#### Architecture Development

##### Overview

This study used The Open Group Architecture Framework (TOGAF) to design the architecture of an integrated PHR system in Indonesia. On the basis of previous studies, TOGAF provides a complete process and methodology to develop architecture [31]. Moreover, TOGAF is the most suitable architectural framework for application in the health sector as it provides a complete architectural development process and can be adapted to the health sector [32,33]. The TOGAF referred to in this study is the TOGAF version 9.2. The Architecture Development Method in the TOGAF can be modified to suit specific needs [34]. In this study, the scope of architectural development was to design information system architecture. Therefore, the TOGAF components needed comprised architecture vision, business architecture, application architecture, data architecture, and technology architecture as required components in designing information system architecture [35].

##### Architecture Vision

According to the TOGAF 9.2, an architectural vision is a brief description of the target architecture that describes the business value and changes that will result from successful implementation. Architectural vision serves as a vision and boundary in the development of a more detailed architecture [34]. The value that is expected to be provided by the integrated PHR is a complete medical history by allowing patients to obtain medical information from different health facilities. Integrated PHRs can also minimize unnecessary health examinations as patients can share their medical history with their physicians so that physicians have information about previous examinations that have been carried out by patients. In addition, the integrated PHR facilitates communication between patients and physicians and helps patients with administrative activities such as registration, appointments, and payment of medical expenses. Integrated PHRs can also help patients manage health outside the health care environment, such as tracking food consumption and physical activity according to the patient’s needs and health conditions.

On the basis of the user requirements and literature review, we formulated architecture principles for integrated PHRs in Indonesia following the TOGAF 9.2 (Table 2). These comprise business, data, application, and technology principles [34]. The business principles comprise information management as everybody’s business, business continuity, service orientation, compliance with the law, and patient-centeredness. Data principles comprise data being an asset, shared, and accessible; common vocabulary and data definitions; and data security. Application principles comprise technological independence and ease of use as well as functionality completeness. The technology principles comprise interoperability and ease of access.

**Table 2:** Architecture principles.

Domain and principle		Description	References
**Business**			
	Information management is everybody’s business	To support health services, health organizations and patients need to be involved in managing information on the PHR^a^.	The Open Group [34] Harahap et al [36]
	Business continuity	The PHR has an optional function that allows users to use it with a poor internet connection.	The Open Group [34] Harahap et al [36]
	Service orientation	The services provided by the PHR are integrated with health care activities in Indonesia.	The Open Group [34] Harahap et al [36]
	Compliance with the law	The PHR needs to comply with all applicable laws, policies, and regulations in Indonesia.	The Open Group [34] Harahap et al [36]
	Patient-centeredness	The PHR is designed for patients to have health information from various sources.	The Open Group [34] Harahap et al [36]
**Data**			
	Data are an asset	The data on the PHR could assist the decision-making of health care providers or patients in managing their health.	The Open Group [34] Harahap et al [36]
	Data are shared	Data can be shared between patients and health care providers.	The Open Group [34] Harahap et al [36]
	Data are accessible	Access to accurate data is needed to improve quality and efficiency in the management of patient health.	The Open Group [34] Harahap et al [36]
	Common vocabulary and data definition	The data on the PHR must have the same definition across health organizations to allow for data sharing.	The Open Group [34] Harahap et al [36]
	Data security	A security mechanism is needed to protect the data stored or exchanged on the PHR.	Harahap et al [5] The Open Group [34] Harahap et al [36]
**Application**			
	Technology independence	The PHR can be integrated with various applications on different platforms.	The Open Group [34] Harahap et al [36]
	Functional completeness	The PHR provides functionalities to support promotive, preventive, curative, and rehabilitative health services in Indonesia.	Harahap et al [5,36]
	Ease of use	The PHR should be easy to use so that users can complete their tasks.	Harahap et al [5] The Open Group [34] Harahap et al [36]
**Technology**			
	Interoperability	The PHR needs to have interoperability standards for sharing data among stakeholders and health information systems.	Harahap et al [5] The Open Group [34] Harahap et al [36]
	Ease of access	The PHR needs to be implemented using common technology used by the community to facilitate easy access to information.	Harahap et al [36] Kharrazi et al [37]

^a^PHR: personal health record.

##### Business Architecture

Business architecture defines the business strategy, governance, organization, and key business processes [34]. On the basis of the interviews with participants from health facilities and a review of health regulations in Indonesia, the business process for PHRs can be divided into health care business processes, self–health monitoring business processes, vaccination business processes, and home care business processes. Each of these process flows can be seen in Multimedia Appendix 7. The main parties involved in the PHR are patients, physicians, health facilities, laboratories, the *Palang Merah Indonesia* or Indonesian Red Cross (PMI), BPJS Kesehatan or other private health insurance companies, and pharmacies.

**Figure 3:**
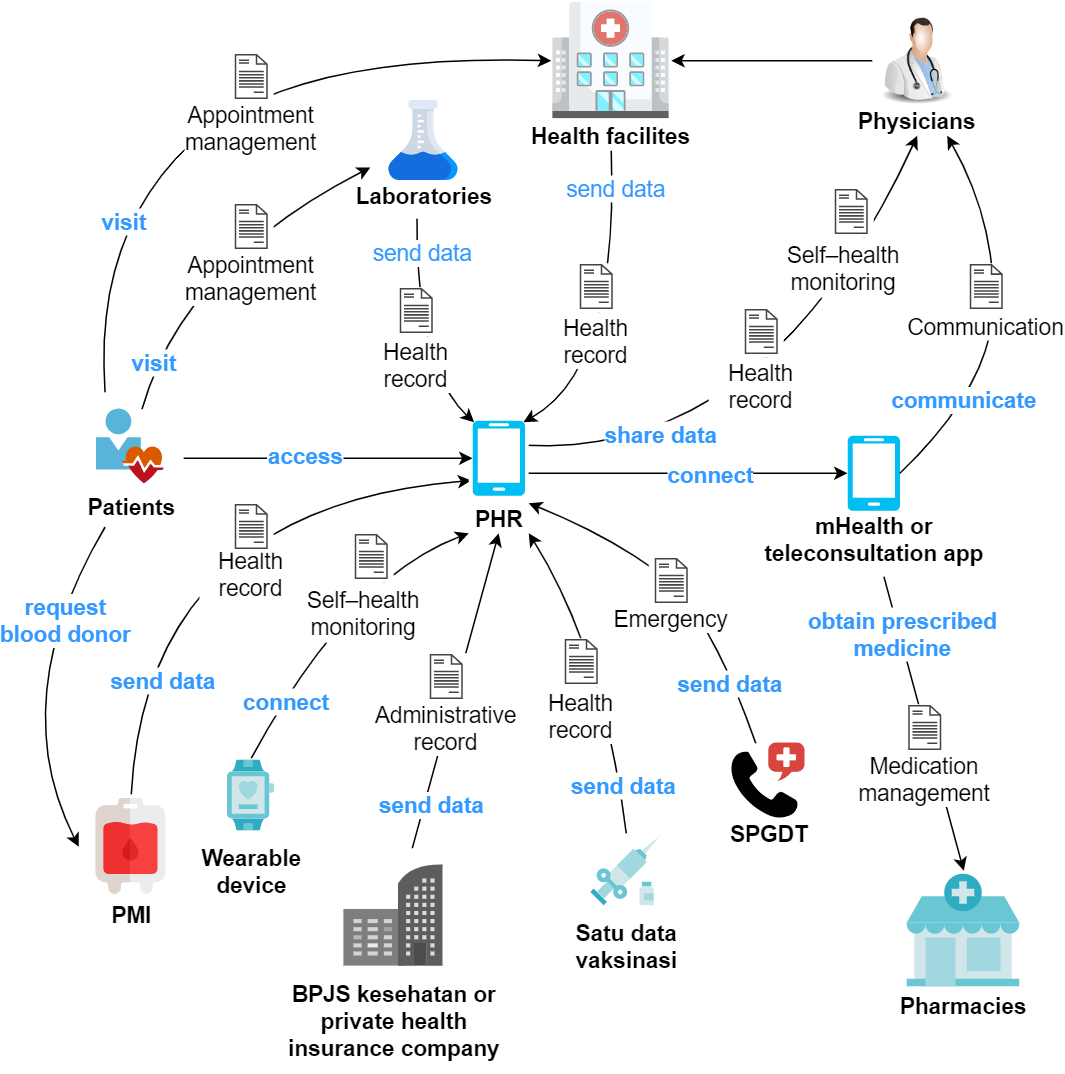
Rich picture of personal health record (PHR) data exchange in the health care process. BPJS: Badan Pelaksana Jaminan Sosial Kesehatan or Social Security Agency for Health; mHealth: mobile health; PMI: Palang Merah Indonesia or Indonesian Red Cross; SPGDT: sistem penanggulangan gawat darurat terpadu or integrated emergency management system.

On the basis of the flow of the process, we added a rich picture to describe the data exchange on PHRs in the health care process (Figure 3). Patients register to make visits to health facilities or laboratories. Patients who need blood donors can also submit a blood donation request to the PMI. Patients can share their health records or personal health monitoring data with their physicians. Patients who have received health services will receive their data in the PHR. Patients can connect the PHR with wearable devices to record personal health monitoring data. Patients can connect the PHR to BPJS Kesehatan or other health insurance companies to obtain their status. Patients can communicate with their physicians or receive their prescribed medicine at pharmacies through the mHealth apps or teleconsultation applications that are connected with the PHR. Moreover, patients can receive vaccination data through the PHR, which is linked to the Ministry of Health vaccine data (*satu data vaksinasi*). The PHR can also connect with an integrated emergency management system (*sistem penanggulangan gawat darurat terpadu*) to obtain the nearest emergency service contacts.

##### Application Architecture

**Figure 4:**
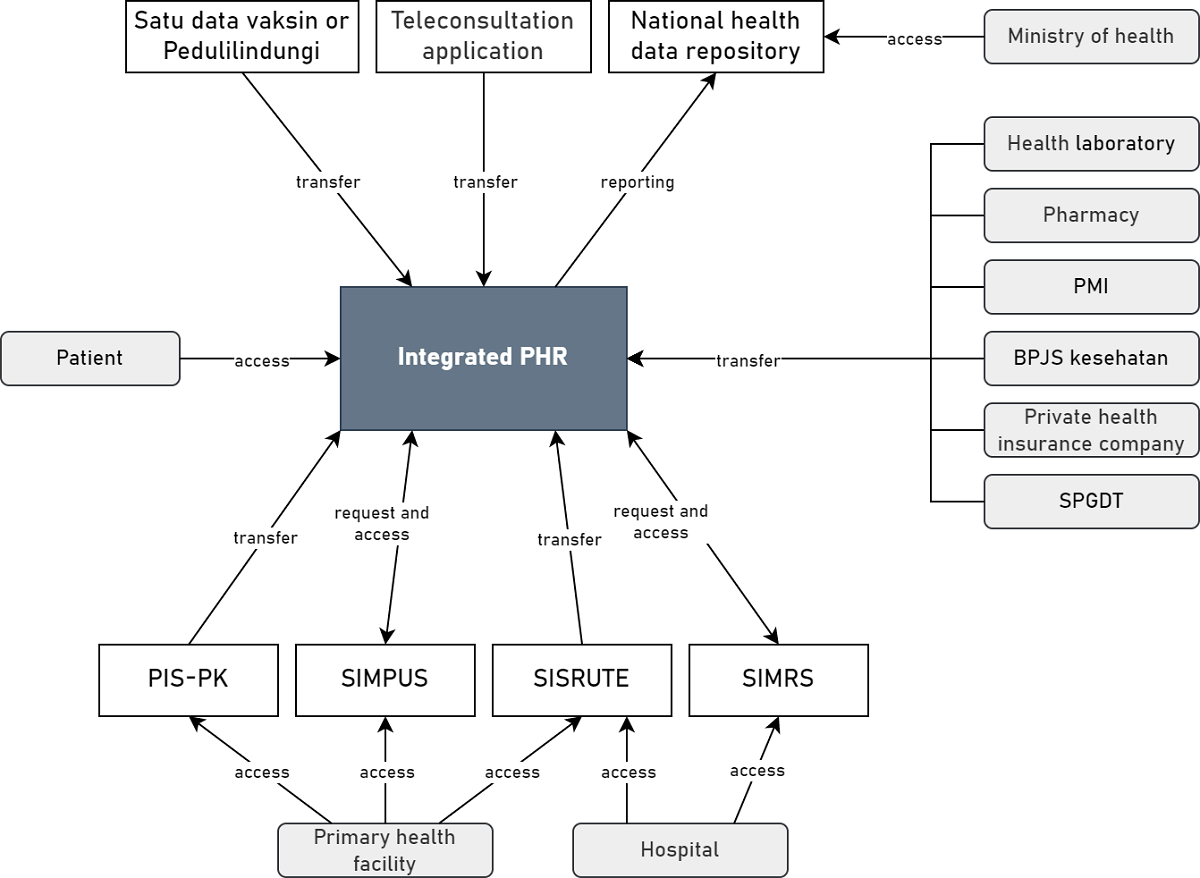
Data exchange of the personal health record (PHR) in Indonesia. BPJS: Badan Pelaksana Jaminan Sosial Kesehatan or Social Security Agency for Health; PIS-PK: Program Indonesia Sehat dengan Pendekatan Keluarga; PMI: Palang Merah Indonesia or Indonesian Red Cross; SIMPUS: sistem informasi puskesmas or primary health care information system; SIMRS: sistem informasi manajemen rumah sakit or hospital information system; SISRUTE: sistem informasi rujukan terintegrasi or referral information system; SPGDT: sistem penanggulangan gawat darurat terpadu or integrated emergency management system.

The TOGAF 9.2 defines application architecture as a blueprint for the applications to be developed, their interactions, and their relationship to the organization’s core business processes [34]. On the basis of the results of interviews and questionnaires, parties that need to be integrated into PHRs are primary health facilities (Puskesmas and clinics), referral health facilities (government and private hospitals), health laboratories, pharmacies, *satu data vaksinasi*, mHealth app or teleconsultation application providers, BPJS Kesehatan, private health insurance companies, and the Ministry of Health. In addition, PHRs need to have the option to connect with wearable devices for tracking health data and family members. On the basis of the results of the business architecture design, other parties that need to be integrated with PHRs are PMI and the integrated emergency management system (*sistem penanggulangan gawat darurat terpadu*). Health facilities are the parties that are most integrated with the information in the PHR. The PHR is integrated with hospital information systems (*sistem informasi manajemen rumah sakit*) in referral health facilities and primary health care information systems (*sistem informasi puskesmas*) in primary health facilities to access health records. In addition to hospital information systems (*sistem informasi manajemen rumah sakit*) and primary health care information systems (*sistem informasi puskesmas*), the PHR is integrated with an integrated referral information system (*sistem informasi rujukan terintegrasi*), which is used by health facilities in Indonesia to access patient referrals. Especially for Puskesmas, the PHR can be integrated with the *Program* Indonesia *Sehat dengan Pendekatan Keluarga* information system. Figure 4 summarizes the integration of the PHR with health information systems or health care providers in Indonesia.

The modules in the PHR comprise health records, administrative records, medication management, communication, appointment management, education, self–health monitoring, security, supporting functions, and emergency. In the health record module, the functionalities comprise a medical summary (results of physical examinations and medical support as well as disease and medication history), referrals, and vaccinations. This module also adds the functions of family planning, home care, and blood donors based on the identification of activities in the business architecture. In the administrative record module, the functionalities comprise patient profiles, health facility profiles, health personnel profiles, health insurance, and payments and billing. In the medication management module, the functionalities comprise medication history, medication reminders, and medication orders. In the communication module, the functionalities comprise messaging. In the appointment management module, the functionalities comprise registration, appointment history, reminders, notifications for appointments, and ambulance services. In the education module, the functionalities comprise health articles containing information on disease problems and health tips. In the self–health monitoring module, the functionalities comprise health data tracking, health dashboards, health calculators, and alert notifications. In the emergency module, the functionalities comprise emergency contacts.

**Figure 5:**
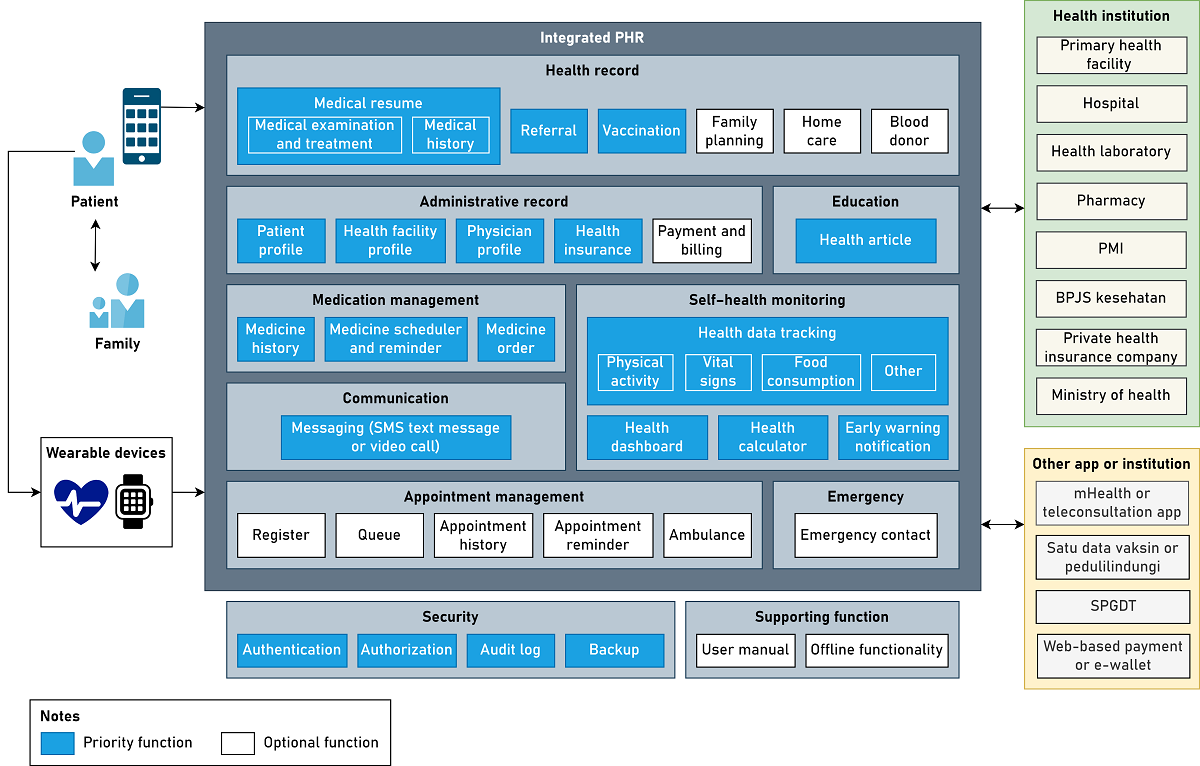
Modules and functionalities of the integrated personal health record (PHR) system in Indonesia. BPJS: Badan Pelaksana Jaminan Sosial Kesehatan or Social Security Agency for Health; mHealth: mobile health; PMI: Palang Merah Indonesia or Indonesian Red Cross; SPGDT: sistem penanggulangan gawat darurat terpadu or integrated emergency management system.

We also designed the modules and functionalities to meet the nonfunctional requirements of PHRs. The modules comprise security and supporting functions. In the security module, the functionalities comprise authentication, authorization, audit logs, and data backup. In the supporting function module, the functionalities comprise user manual and offline functionality. The functionalities that need to be prioritized for implementation in PHRs are functions related to health management (health care, health prevention, and health promotion) and functions to support information security, whereas other functionalities such as functions related to administration and supporting functions are optional. To access the PHR, the platform used is a smartphone as, based on the results of the questionnaire, it is the most widely used platform by the public to access health applications. Figure 5 summarizes the integrated PHR system model in Indonesia.

##### Data Architecture

The TOGAF 9.2 defines data architecture as the logical and physical structure of the organization’s data assets and data management resources [34]. We grouped data in PHRs into 3 data categories comprising master, transaction, and reference data [38,39]. Transaction data relate to data that are recorded every time a transaction occurs, such as medical records, vaccinations, and health referrals. Master data are data that do not change and do not need to be recorded in every transaction, such as patient and health facility data. Reference data are a collection of values or classifications that can be referenced by master and transaction data.

##### Technology Architecture

**Figure 6:**
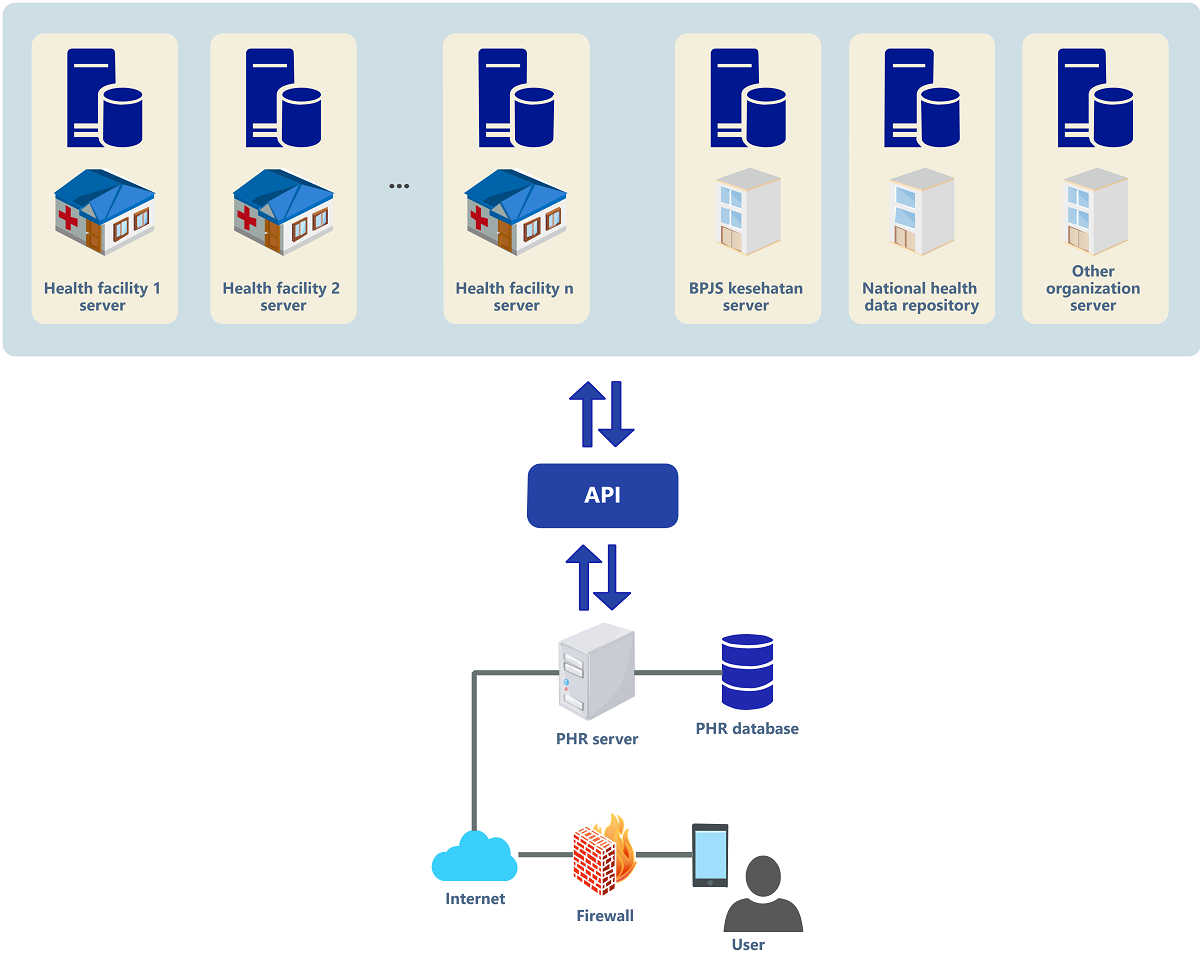
Technology architecture. API: application programming interface; BPJS: Badan Pelaksana Jaminan Sosial Kesehatan or Social Security Agency for Health; PHR: personal health record.

The technology architecture for PHRs is described in the form of a high-level architecture to illustrate the technology required for PHR implementation and integration with other systems (Figure 6). Patients or individuals access the PHR through a mobile app. PHR development uses the React Native cross-platform app development that can be implemented on the Android or iOS platforms. The PHR application is accessed by users via the internet, and a firewall is used for security. The PHR server comprises an application server and a database server. The application server provides access to data for the user, whereas the database server provides the data requested by the application server [40].

An application programming interface (API) is used as an intermediary for interaction between the PHR and other information systems. The type of API used is Fast Healthcare Interoperability Resources (FHIR). FHIR is an international standard recommended by the Ministry of Health to solve the problem of data exchange in health information systems in Indonesia [17]. FHIR is flexible and can be adapted to stakeholder needs, clinical specifications, and health policies. FHIR can be used to manage a single data entity (eg, heart rate), groups of data entities (eg, vital signs, medications, and allergies), or electronic recording systems such as PHRs. Therefore, FHIR is suitable for exchanging data on PHRs as PHRs aim to collect and exchange individual health data [41].

#### Prototype Development

**Figure 7:**
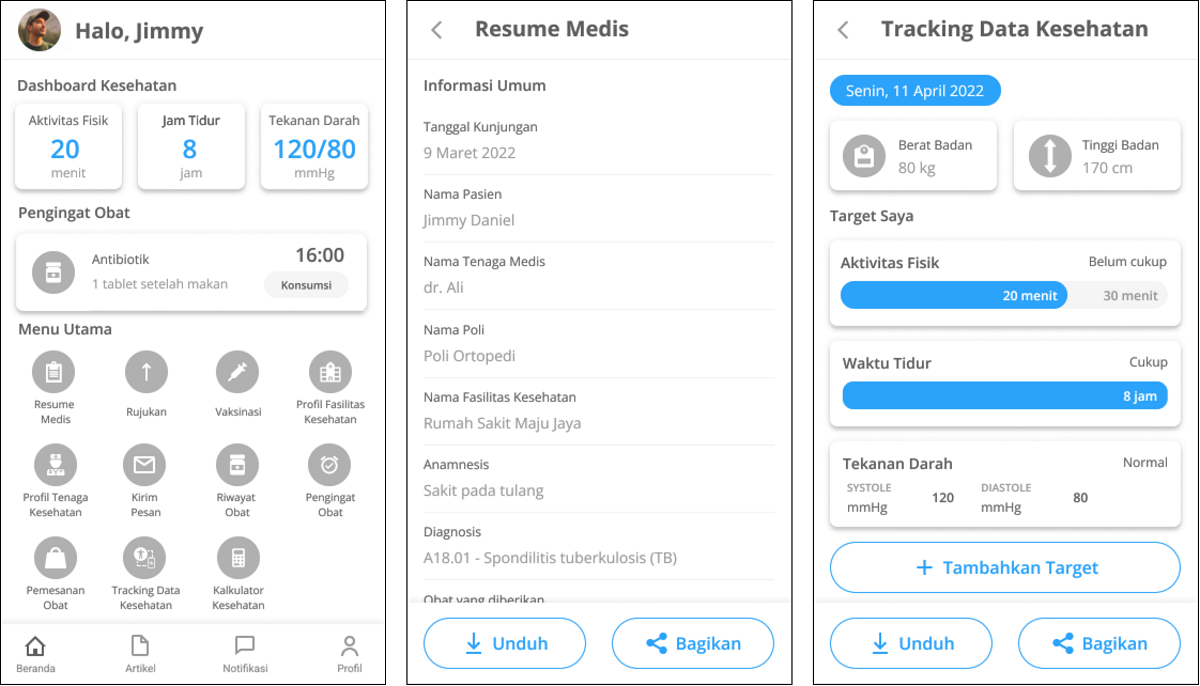
Example of the home page, medical summary, and health data tracking in the patient prototype.

**Figure 8:**
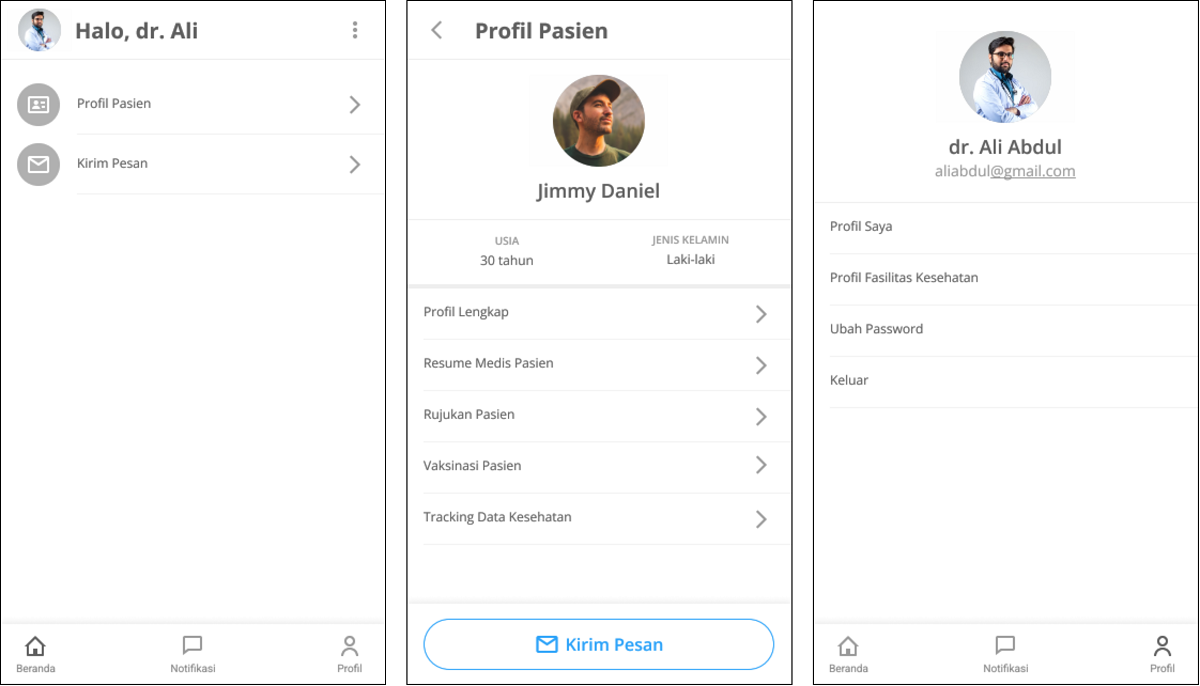
Example of the home page, patient profile, and physician profile from the physician prototype.

The main actors involved in health care and management activities are patients and physicians. Patients are actors who receive health services and play a role in managing their health through applications. Physicians are actors who provide health services to patients. Physicians comprise general practitioners and specialists. The functionalities developed in the prototype design are priority functions defined in the application architecture, which comprise medical summaries, referrals, vaccinations, health facility profiles, physician profiles, patient profiles, messaging, medication history, medication reminders, medication orders, health data tracking, health calculators, health articles, and notifications. An explanation of the design requirements for each function and the actors involved is presented in Table 3. We developed a high-fidelity prototype for a mobile app. Some examples of patient and physician prototype designs are shown in Figures 7 and 8, respectively.

**Table 3:** Design requirements for application prototype.

Function	Description	Actor
Medical summary	View the history of patient visits to health facilities, including detailed examination results	Patient and physician
Referral	View patient referral history and detailed examination results from each patient referral	Patient and physician
Vaccination	View a patient’s vaccination history	Patient and physician
Health facility profile	Search for health facilities and see the nearest health facility	Patient
Health facility profile	View detailed health facility information	Patient and physician
Physician profile	View the profile of physicians who have treated patients	Patient
Physician profile	Manage physician profile	Physician
Patient profile	Manage patient profiles, including adding family members, connecting with BPJS^a^ Kesehatan or other health insurance companies, and connecting with wearable devices	Patient
Patient profile	View a list of patients who have been treated	Physician
Messaging	View lists and details of messages between patient and physician	Patient and physician
Medication history	View a list of past or current medications	Patient
Medication reminder	Manage reminders to take medication	Patient
Medication order	Obtain the prescribed medicine at the pharmacy	Patient
Health data tracking	Record health monitoring data such as physical activity, food consumption, and others	Patient
Health data tracking	View the health monitoring dashboard that has been created by the patient	Patient and physician
Health calculator	Perform patient health calculations such as BMI	Patient
Health article	Read health articles, such as information about disease problems or health tips	Patient
Notification	Receive notifications such as incoming messages, documents sent, or reminders to perform certain activities	Patient and physician

^a^BPJS: Social Security Agency for Health or Badan Pelaksana Jaminan Sosial Kesehatan.

### Artifact Evaluation

#### Respondent Demographics

To evaluate the architectural design and application prototype, we conducted interviews with IT or eHealth experts. Interviews were conducted with 6 respondents: 1 (17%) respondent from the Ministry of Health, 1 (17%) academician, 1 (17%) respondent from a government hospital, 1 (17%) respondent from a private hospital, and 2 (33%) health application vendors. Interviews were conducted from April 5, 2022, to April 8, 2022, with an interview duration of 40 to 60 minutes. The information of the respondents is presented in Table 4.

**Table 4:** Respondent demographics.

Respondent code	Sex	Role	Work experience (years)
E1	Male	Health application vendor	1-5
E2	Female	Academician	1-5
E3	Male	IT management in the Ministry of Health	>10
E4	Male	IT management in a government hospital	>10
E5	Male	IT management in a private hospital	>10
E6	Male	Health application vendor	>10

#### Architecture Evaluation

The evaluation was carried out to assess the suitability of the integrated PHR design for the needs of health services in Indonesia (Table 5). All respondents (6/6, 100%) stated that the architectural vision and business architecture in the PHR architectural design described the needs of health services in Indonesia. Regarding the integration in the application architecture, there were several recommendations from respondents regarding parties that needed to be integrated with the PHR but were not described in the application architecture design. Other parties that need to be integrated with the PHR include billing gateways for the payment function (respondent E1) and the Directorate General of Population and Civil Registration for patient identity (respondents E2 and E3). A respondent (E4) suggested adding a health screening function to the PHR. Another respondent (E3) suggested making the messaging and medication order functions optional as they can be connected with other applications. Regarding data architecture, respondent E6 commented that the data architecture was sufficient as long as there was an explanation of the data source in the PHR. For security, respondents suggested more options for authentication methods, such as biometrics (respondent E1) and face recognition (respondent E5). Regarding technology architecture, 100% (6/6) of the respondents stated that the use of an API was a suitable solution for integration between PHRs and other applications in Indonesia. Respondent E3 commented that FHIR was the right type of API to use for PHR implementation. For security and privacy needs, respondents also agreed with the use of firewalls in the technology architecture.

**Figure 9:**
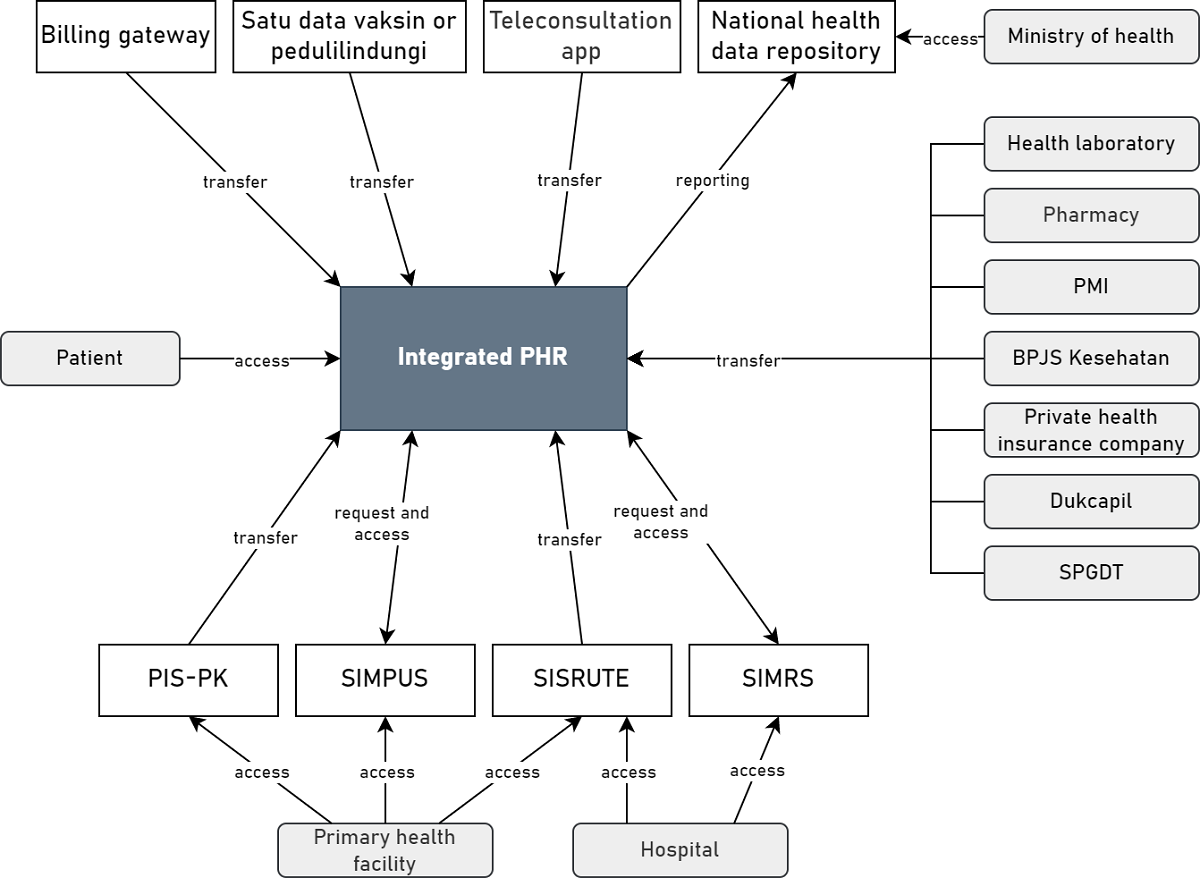
Design improvements to the data exchange in the personal health record (PHR) in Indonesia. BPJS: Badan Pelaksana Jaminan Sosial Kesehatan or Social Security Agency for Health; Dukcapil: Directorate General of Population and Civil Registration; PIS-PK: Program Indonesia Sehat dengan Pendekatan Keluarga; PMI: Palang Merah Indonesia or Indonesian Red Cross; SIMPUS: sistem informasi puskesmas or primary health care information system; SIMRS: sistem informasi manajemen rumah sakit or hospital information system; SISRUTE: sistem informasi rujukan terintegrasi or referral information system; SPGDT: sistem penanggulangan gawat darurat terpadu or integrated emergency management system.

**Figure 10:**
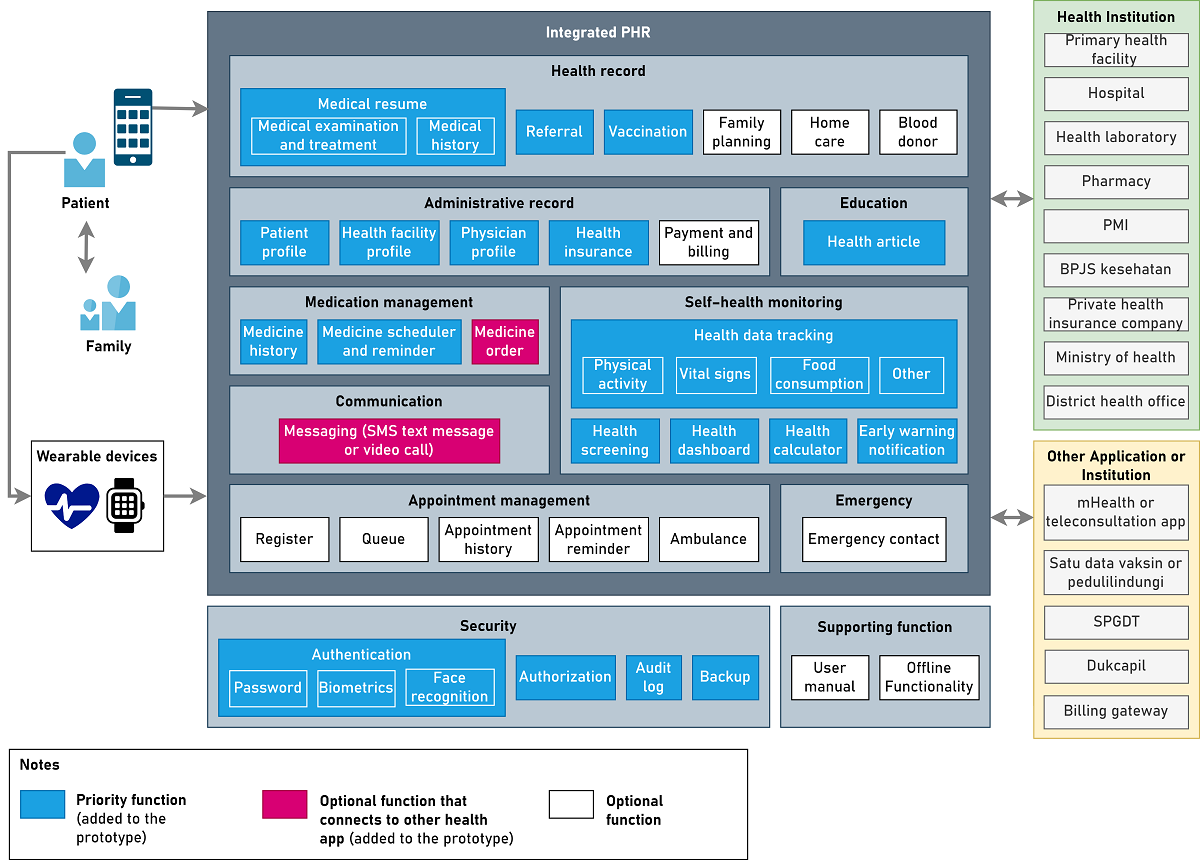
Design improvements to the modules and functionalities of the integrated personal health record (PHR) system in Indonesia. BPJS: Badan Pelaksana Jaminan Sosial Kesehatan or Social Security Agency for Health; Dukcapil: Directorate General of Population and Civil Registration; mHealth: mobile health; PMI: Palang Merah Indonesia or Indonesian Red Cross; SPGDT: sistem penanggulangan gawat darurat terpadu or integrated emergency management system.

On the basis of the evaluation with IT and eHealth experts, improvements were made by adding billing gateways and Directorate General of Population and Civil Registration to the application architecture (Figure 9). Improvements were also made to the modules and functionality of the integrated PHR system in Indonesia in application architecture (Figure 10). In the medication management module, the medication order function was changed from a priority function to an optional function that can be connected with mHealth apps or teleconsultation applications in Indonesia. In the communication module, the messaging function was also changed from a priority function to an optional function that can be connected with mHealth apps or teleconsultation applications in Indonesia. Improvements were also made by adding a health screening function to the self–health monitoring module. In the security module, authentication methods were added, including passwords, biometrics, and face recognition. Descriptions of each module and functionality in the PHR are summarized in Multimedia Appendix 8. Improvements to the data architecture were made to add health screening data to the transaction data category. Data categories with data groups and descriptions in the PHR are described in Multimedia Appendix 9.

**Table 5:** Summary of personal health record (PHR) architecture evaluation results.

Architecture component	Evaluation criteria	Evaluation results	Respondent
Architecture vision	Suitability for the health services in Indonesia	It is sufficient to describe the needs of health services in Indonesia	E1, E2, E3, E4, E5, and E6
Business architecture	Conformity with the process of health services in Indonesia	It is sufficient to describe the needs of health services in Indonesia	E1, E2, E3, E4, E5, and E6
Application architecture	Integration with health information systems or other parties	Integration with billing gateway	E1
Application architecture	Integration with health information systems or other parties	Integration with Dukcapil^a^	E2 and E3
Application architecture	Completeness of functionality	Messaging and medication orders as optional functions	E3
Application architecture	Completeness of functionality	Addition of health screening function	E4
Application architecture	Completeness of functionality	Addition of authentication method options such as biometrics and face recognition	E1 and E5
Data architecture	Data requirements and completeness	Data architecture is sufficient as long as there is an explanation of the data source in the PHR	E6
Technology architecture	Technology requirements for PHR implementation	The architecture already describes the technology requirements for PHR implementation	E1, E2, E3, E4, E5, and E6

^a^Dukcapil: Directorate General of Population and Civil Registration.

#### Prototype Evaluation

**Figure 11:**
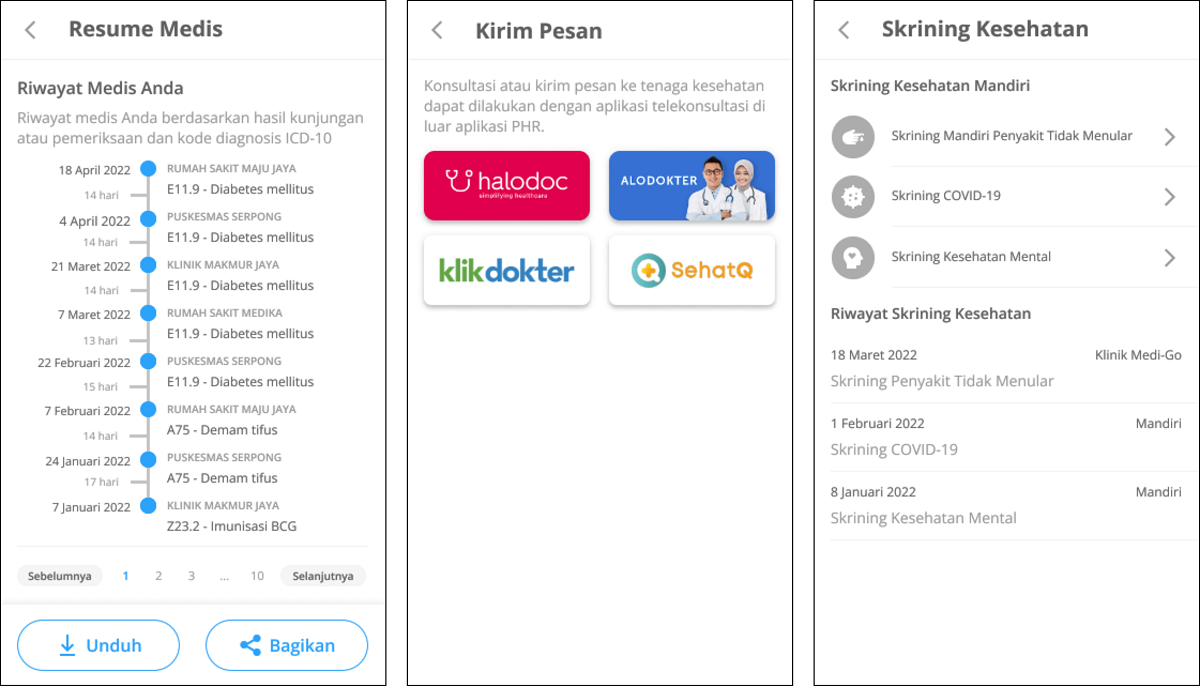
Examples of prototype improvements.

The evaluation of the prototype design resulted in suggestions for improvements related to the main functions developed in the prototype design (Table 6). Suggestions for improvements that need to be made were the functions of medical summary, referral, vaccination, physician profile, messaging, medication history, medication reminder, medication order, health data tracking, notification, and patient profile. In the medical summary, referral, and vaccination functions, a respondent (E3) suggested adding a patient identification number. Some examples of the prototype improvements are shown in Figure 11.

In the medical summary function, suggestions for improvement were the addition of the patient’s overall medical history (respondent E3) and the addition of the patient’s medical record number (respondent E5) to the medical summary details. The addition of the patient’s overall medical history aimed to make it easier for patients to view their complete medical history without having to look at the details of each medical summary one by one. The patient’s medical record number was intended to be the patient’s identity number at the health facility.

In the referral function, suggestions for improvement were the addition of information on the actions given before the patient was referred (respondent E1) and the reason for the patient being referred (respondent E2) in the referral details. Other suggestions for improvement were the addition of information on the type of referral, such as back referral (respondent E2). This information is needed for the continuous treatment of patients as health workers who treat patients need to know the complete condition of the patient.

In the vaccination function, suggestions for improvement included adding other types of vaccinations apart from COVID-19 (respondents E1, E2, E3, and E4). Additional types of vaccinations are needed so that this function can be used not only during the COVID-19 pandemic but also after it ends. In addition, in the vaccination function, it was recommended that the patient be able to view the vaccination history of family members (respondent E1).

In the physician profile function, the suggestion for improvement was changing the physician’s ID to the *Surat Izin Praktik* number (respondents E1, E2, and E4) in the detail of the physician profile as the *Surat Izin Praktik* number is the standard numbering used for every health facility in Indonesia. Another suggestion for improving this function was the deletion of information to view the recommendations of the nearest physician (respondents E3 and E5). This is because, in implementation, it will be difficult to obtain updated information on physicians’ availability around the patient’s location.

In the messaging function, the suggestions for improvement were to link this function to health applications or teleconsultation applications that already exist in Indonesia. The goal is to facilitate the implementation of the PHR application as other applications can handle the messaging function. This respondent (E3) also suggested doing the same for the medication order function.

In the medication history function, a suggestion for improvement was given by a respondent (E1) to add medication categories, such as generic or patent. The respondent also gave suggestions on the medication reminder function to add information on how to take the medication. Another suggestion for improvement in medication reminders was the need to add information on whether the medication should be fully consumed (respondent E5).

**Table 6:** Summary of prototype evaluation results.

Actor and function		Evaluation result
**Patient**		
	Medical summary	Add a medical summary dashboard to view the patient’s overall medical history (respondent E3) Add the patient’s NIK^a^ number (respondent E3) Add the patient’s medical record number to the medical summary details (respondent E5)
	Referral	Add reason for referral (respondent E2) Add medication treatment information to referral details (respondent E2) Add referral type (respondent E2) Add the patient’s NIK number (respondent E3)
	Vaccination	Add vaccine category other than COVID-19 vaccine (respondents E1, E2, E3, and E4) Add the patient’s NIK number (respondent E3)
	Physician profile	Change physician ID to SIP^b^ number (respondents E1, E2, and E4) Delete nearest physician recommendations (respondents E3 and E5)
	Messaging	Link to other health applications (respondent E3)
	Medication history	Add patent or generic medication category (respondent E1)
	Medication reminder	Add the way of taking the medication (respondent E1) Add information on whether the medication must be fully consumed or not (respondent E5)
	Medication order	Link to other health applications (respondent E3)
	Health data tracking	Add weekly health data tracking dashboard (respondent E1) Add sample health data tracking input page (respondent E5)
	Notification	Display changes to distinguish read from unread notifications (respondent E1)
	Patient profile	Add other security options such as biometrics (respondent E1) Add blood type to patient profile details (respondent E2) Add marital status to patient profile details (respondent E5)
	Other	Add log-in or registration options with Gmail (respondent E1) Add health screening function (respondent E4)
**Physician**		
	Patient profile	Add a dashboard to view patients who need to be responded to (respondent E1) Add menu of patient health screening results (respondent E4)
	Notification	Add sample notification for referral (respondent E2)
	Physician profile	Delete the function to view the health facility profile on the physician profile (respondents E4 and E5)

^a^NIK: Nomor Induk Kependudukan (patient identification number).

^b^SIP: Surat Izin Praktik number (standard numbering used for every health professional or health care provider [physician, nurse, and midwife] in Indonesia).

In the health data tracking function, suggestions for improvement were the need for a weekly health dashboard to observe trends in patient health tracking (respondent E1). Another suggestion was to add a sample data input page for this function (respondent E6). In the notification function, the suggestion for improvement was the need for display adjustments to indicate the difference between read and unread notifications (respondent E1).

In the patient profile function, suggestions for improvement included the need for security options other than passwords, such as biometrics (respondent E1). In the detailed patient profile, additional information was needed, such as blood type (respondent E2) and marital status (respondent E5). In addition to the functions that have been discussed, respondent E4 gave suggestions to add a health screening function to the PHR, including independent health screening or health screening in primary health facilities.

In the evaluation of the prototype design for the physician, suggestions for improvement were the addition of a dashboard to see patients who need to be responded to (respondent E1) and the addition of patient health screening results following the suggestions for the prototype design for patients (respondent E4). Another improvement suggestion was the addition of a notification example for referral data that are sent from patients to the physician (respondent E2). Furthermore, for the user profile function, suggestions for improvement were to remove unnecessary information, such as the profile function of health facilities (respondents E4 and E5).

## Discussion

### Principal Findings

This study designed an integrated PHR system architecture in Indonesia and an application prototype. In Indonesia, there are various mHealth apps or teleconsultation applications, such as AloDokter, Halodoc, and Mobile JKN. AloDokter and Halodoc are connected to health care providers, mostly private clinics and hospitals, whereas Mobile JKN is for JKN patients in primary health facilities such as Puskesmas and clinics. However, the exchange of health information in these applications is one-way, from the health facility or physician to the patient. Previous research on the adoption of PHRs has also found that health facilities in Indonesia generally do not provide web-based access to patients’ health records [36]. This study provides a PHR model that is connected with various health care providers and is integrated into routine health care practice in Indonesia.

A review conducted by Hoque et al [42] stated that most health applications or mHealth research in low- and middle-income countries do not follow the design science approach. The DSR approach informs how artifact validation is carried out so that it can provide evidence that the designed artifact is useful and meets the users’ requirements [21]. This study uses a DSR approach with evaluations carried out by IT or eHealth experts so that the PHR design follows the practice of health services in Indonesia.

The architecture development based on the TOGAF can describe the need for integration into the PHR by describing who are the stakeholders involved in the PHR. Architecture development using the TOGAF can help align business processes, data, applications, and IT infrastructure [43]. The architectural design developed in this study covers the provision of essential or basic routine health services that always exist in the community, such as health examinations, disease treatment, and immunization [44]. The development of this PHR can also support national health priorities and the Germas program, which prioritizes promotive and preventive health efforts, especially for the prevention and control of noncommunicable diseases [45].

The application prototype in this study was developed as a mobile app. The increasingly widespread use of smartphone apps in the community and the ease of access to smartphones encourage the adoption of PHRs on mobile devices, or mobile PHRs [37]. In Indonesia, the number of smartphone users has reached 167 million, or 89% of the total population [46]. The results of the questionnaire in this study on the use of health applications section also showed that smartphones were the most popular devices for patients or individuals to access health applications in Indonesia. Smartphones also offer unique features such as a camera, GPS, and touch screen that can be used to extend the usefulness of mobile PHR, such as scanning and importing paper documents, recording certain symptoms, creating videos, or scanning bar codes for medical purposes [37].

### Implications

As explained in the Introduction section, previous research on PHR design in low- and middle-income countries [6-9] has involved users in exploring the needs and usability of the PHR design. However, they did not explain the integration of the PHR with other health applications. Although there is a study describing PHR integration using the distributed PHR model, this study did not involve users or stakeholders in designing the model [10]. Our study complements the gaps in previous studies in low- and middle-income countries by designing an integrated PHR in Indonesia, which involves related stakeholders in requirement gathering and evaluation.

The theoretical implications of this research are the contribution to the field of PHR research by presenting design science as an approach for designing an integrated PHR system for a low- or middle-income country context that takes into account the specific characteristics of the Indonesian health care system. By using DSR, it can be ensured that the PHR model developed is based on scientific theory and methods. In addition, the DSR approach helps researchers understand existing health care systems and the needs of various stakeholders, such as patients, health care providers, and health regulators, in developing PHRs. DSR then includes evaluating the proposed PHR model, which can help identify any issues and make necessary improvements so that the designed PHR can follow the health system in Indonesia.

We developed architecture and application prototypes based on health systems in Indonesia, which comprise routine health services, including disease treatment and health examinations, as well as promotive and preventive health efforts. In addition, in this study, there are health referral functions that have not been discussed in the previous review of PHR functionality [5] and other studies in low- and middle-income countries [6-10]. This referral function is needed as the health system in Indonesia has a tiered referral program that should be followed by JKN patients [13].

The practical implication of this study is that this research is expected to be a guide for health regulators, health facilities, or health application vendors in designing an integrated PHR system in Indonesia. The architectural design in this study can provide an overview to integrate the PHR into the health services process in Indonesia, information about parties that need to be integrated into the PHR, and technology that can be used for PHR implementation. The prototype design in this study provides a guideline to implement PHR functions that focus not only on health care but also on disease prevention and health promotion.

### Conclusions

The architecture design of the integrated PHR system in Indonesia refers to the TOGAF version 9.2, which is divided into 5 components: architecture vision, business architecture, application architecture, data architecture, and technology architecture. We developed a high-fidelity prototype for patients and physicians. The functionalities that were implemented were the priority functions defined in the architecture. The architecture evaluation stated that the architecture design had already described the needs and processes of health services in Indonesia as well as the technology needed for implementation. Improvements were made to the application architecture and data architecture to add the stakeholders that need to be integrated and the required functionality to the PHR. Prototype evaluation resulted in adding the necessary information to the functions that were developed, such as linking the medication order and messaging functions to the teleconsultation application and adding a health screening function. The limitation of this research is that the evaluation only focused on assessing the suitability of the integrated PHR model for the needs of health programs in Indonesia from the perspective of IT or eHealth experts. Future studies should be conducted to evaluate the prototype PHR from the perspective of patients and physicians as the primary users of the PHR.

## Appendix

Multimedia Appendix 1 Interview guide.

Multimedia Appendix 2 COREQ (Consolidated Criteria for Reporting Qualitative Research) checklist.

Multimedia Appendix 3 Interview respondents.

Multimedia Appendix 4 Health application use.

Multimedia Appendix 5 Functionality codes.

Multimedia Appendix 6 Summary of the personal health record module and functionality based on the requirements of health organizations and patients.

Multimedia Appendix 7 Process flows in the personal health record.

Multimedia Appendix 8 Module and functionality in the personal health record.

Multimedia Appendix 9 Data categories with data groups and descriptions in the personal health record.

## Abbreviations

API: application programming interface
BPJS Kesehatan: Badan Pelaksana Jaminan Sosial Kesehatan or Social Security Agency for Health
COREQ: Consolidated Criteria for Reporting Qualitative Research
DSR: design science research
FHIR: Fast Healthcare Interoperability Resources
GH: General hospital
HR: health regulator
JKN: Jaminan Kesehatan Nasional or national health insurance
mHealth: mobile health
PH: private hospital
PHC: primary health care
PHR: personal health record
PMI: Palang Merah Indonesia or Indonesian Red Cross
Puskesmas: pusat kesehatan masyarakat or primary health centers
TOGAF: The Open Group Architecture Framework
VDR: vendor
