# Supplementary material for: Integrated Personal Health Record in Indonesia: Design Science Research Study
Source: JMIR Med Inform. 2023 Mar 14;11:e44784. doi: 10.2196/44784 (PMC10131695; doi:10.2196/44784)
Supplement: Multimedia Appendix 1 [file medinform_v11i1e44784_app1.docx]

## **Interview Guide**

1. Interview Guide for Health Facility Management/Ministry of Health/BPJS *Kesehatan*/Health Application Vendor
2. What health information systems or applications have currently been developed by your organization? Who are the users of the applications? What features are in those health applications?
3. Is there a PHR application or application that patients can use to access medical resumes in health facilities? (Examples: lab results, diagnosis, medical history, clinical notes)
4. What functional requirements or features need to be implemented in the future?
5. How is the integration of PHR with other stakeholders?
6. What are the concepts and mechanisms of data integration or exchange between PHR and other health applications?
7. How does your organization guarantee the security and privacy of data?
8. How does your organization guarantee the quality of data?
9. How does your organization guarantee the ease of use of health applications?
10. What national regulations or international standards can be used as references for your organization in developing health applications (including data standards, security, privacy, interoperability, etc.)?
11. What are the challenges in implementing the PHR application or other current health applications in Indonesia? What are your recommendations for the implementation of PHR in the future?
12. Interview Guide for Health Professionals (Physician/Dentist/Nurse)
13. What health information systems or applications are you currently using? What features do you use in the health application?
14. Is there a PHR application or application that you can use to exchange medical data (e.g. lab results, diagnosis, medical history, clinical notes) with patients?
15. What functional requirements or features need to be implemented in the future?
16. What is the role of health facilities in supporting the implementation of PHR or existing health applications?
17. What are the challenges that you faced in using PHR or current health applications?
18. What are your recommendations for the implementation of PHR in the future?
