# Supplementary material for: Integrated Personal Health Record in Indonesia: Design Science Research Study
Source: JMIR Med Inform. 2023 Mar 14;11:e44784. doi: 10.2196/44784 (PMC10131695; doi:10.2196/44784)
Supplement: Multimedia Appendix 3 [file medinform_v11i1e44784_app3.docx]

## **Multimedia Appendix 3. Interview Respondents**

| **Organization Code** | **Province** | **Respondent’s Position/Department** | **Number of Respondents** |
| --- | --- | --- | --- |
| GH1 | DKI Jakarta | Hospital director | 1 |
| GH2 | DKI Jakarta | Head of IT | 1 |
| GH3 | Bali | Head of IT | 1 |
| GH4 | DKI Jakarta | Physician | 1 |
|  |  | Nurse | 3 |
| GH5 | DI Yogyakarta | Head of IT | 1 |
| GH6 | Bali | Head of IT | 1 |
|  |  | Dentist | 1 |
| GH7 | DKI Jakarta | Physician | 1 |
| GH8 | Sulawesi Selatan | Physician | 1 |
| GH9 | DKI Jakarta | Head of IT | 1 |
| PH1 | Banten | Head of IT | 1 |
| PH2 | DKI Jakarta | Head of IT | 1 |
|  |  | Physician | 1 |
| PH3 | Jawa Barat | Head of IT | 1 |
| PH4 | DKI Jakarta | Hospital director | 1 |
| PH5 | Jawa Barat | Dentist | 1 |
| PH6 | DKI Jakarta | Physician | 2 |
| PHC1 | Jawa Barat | Head of IT | 1 |
| PHC2 | Jawa Barat | Head of IT | 1 |
| PHC3 | DKI Jakarta | Head of IT | 1 |
| PHC4 | DKI Jakarta | Clinic owner | 1 |
| PHC5 | Jawa Barat | Head of IT | 1 |
| PHC6 | Riau | Head of IT | 1 |
| PHC7 | DKI Jakarta | Head of IT | 1 |
| PHC8 | DKI Jakarta | Head of IT | 1 |
| PHC9 | DKI Jakarta | Head of IT | 1 |
| PHC10 | DKI Jakarta | Head of IT | 1 |
| HR1 | DKI Jakarta | Member of data and information center | 2 |
| HR2 | DKI Jakarta | Head of IT planning strategy | 1 |
| VDR1 | Jawa Barat | Health application vendor | 1 |
| VDR2 | DKI Jakarta | Health application vendor | 1 |
| VDR3 | DKI Jakarta | Health application vendor | 1 |
