# Supplementary material for: Integrated Personal Health Record in Indonesia: Design Science Research Study
Source: JMIR Med Inform. 2023 Mar 14;11:e44784. doi: 10.2196/44784 (PMC10131695; doi:10.2196/44784)
Supplement: Multimedia Appendix 4 [file medinform_v11i1e44784_app4.docx]

## **Multimedia Appendix 4. Health Application Usage**

| **Question** | **Attribute** | **Number of respondents (%)** |
| --- | --- | --- |
| Use of health applications to seek treatment or read health information | Yes | 709 (70,06%) |
|  | No | 303 (29,94%) |
| The questions below are asked if the previous question was answered “Yes” | | |
| Health application used (can be answered more than one option) | Alodokter | 245 (34,41%) |
|  | Halodoc | 509 (71,49%) |
|  | MobileJKN | 129 (18,12%) |
|  | PeduliLindungi | 422 (59,27%) |
|  | KlikDokter | 56 (7,87%) |
|  | Lainnya | 48 (6,74%) |
| Platforms to access health applications (can be answered more than one option) | Computer | 105 (14,75%) |
|  | Notebook | 76 (10,67%) |
|  | Tablet | 32 (4,49%) |
|  | Smartphone | 694 (97,47%) |
| Health application usage period | < 1 year | 331 (46,69%) |
|  | 1-3 years | 317 (44,71%) |
|  | 3-5 years | 42 (5,92%) |
|  | > 5 years | 19 (2,68%) |
| Frequency of using health applications in the last 6 months | 1-5 times | 560 (78,98%) |
|  | 6-10 times | 85 (11,99%) |
|  | > 10 times | 64 (9,03%) |
| Features used in the health application (can be answered more than one option) | View medical records, such as diagnoses, lab results, or medical history | 263 (37,09%) |
|  | View information on health facilities or physician profile | 385 (54,30%) |
|  | Order medicine | 263 (37,09%) |
|  | Chat or online consultation with a physician | 374 (52,75%) |
|  | Make an appointment with a physician | 132 (18,62%) |
|  | Search and read health education content | 345 (48,66%) |
|  | Input personal health data, such as physical activities and food consumption | 70 (9,87%) |
|  | Other | 23 (3,24%) |
| Reasons for using health applications (can be answered by more than one option) | Recommendations from friends, family, or relatives | 209 (29,48%) |
|  | Cheaper medical costs | 95 (13,40%) |
|  | Faster and easier access to health services | 561 (79,13%) |
|  | The requirement from a health facility to seek a treatment | 104 (14,67%) |
|  | Other | 56 (7,90%) |
| Problems encountered when using health applications (can be answered by more than one option) | Poor internet connection | 97 (13,68%) |
|  | Concerns about data privacy | 224 (31,59%) |
|  | Incomplete information or features on the health application | 113 (15,94%) |
|  | Difficulty to understand medical terminologies | 41 (5,78%) |
|  | Unattractive user interface | 23 (3,24%) |
|  | Health applications often experienced errors/crashes/down | 74 (10,44%) |
|  | Never encounter any problems | 117 (16,50%) |
|  | Other | 20 (2,82%) |
| Organizations that should be integrated or connected to health applications (can be answered by more than one option) | Hospital | 566 (79,83%) |
|  | Puskesmas or Clinic | 436 (61,50%) |
|  | Health laboratory | 365 (51,48%) |
|  | Ministry of Health | 274 (38,65%) |
|  | BPJS Kesehatan | 437 (61,64%) |
|  | Other | 26 (3,67%) |
| The most important main component of a health application | Functionality completeness | 443 (62,48%) |
|  | Ease of use | 536 (75,60%) |
|  | Integration or interoperability | 484 (68,27%) |
|  | Security and privacy | 492 (69,39%) |
|  | Performance | 321 (45,28%) |
|  | Other | 18 (2,54%) |
