# Supplementary material for: Integrated Personal Health Record in Indonesia: Design Science Research Study
Source: JMIR Med Inform. 2023 Mar 14;11:e44784. doi: 10.2196/44784 (PMC10131695; doi:10.2196/44784)
Supplement: Multimedia Appendix 5 [file medinform_v11i1e44784_app5.docx]

## **Multimedia Appendix 5. Functionalities Code**

| **Functionality Code** | **Description** |
| --- | --- |
| HealthRecord_01 | View the results of the medical examination, such as lab tests, radiology, etc. |
| HealthRecord_02 | View medical history |
| HealthRecord_03 | View vaccination history |
| HealthRecord_04 | View health referral history |
| HealthRecord_05 | Sharing information regarding medical examination and history with physicians |
| AdministrativeRecord_01 | Manage patient demographic data, such as name, gender, date of birth, and contact number |
| AdministrativeRecord_02 | View health facility information, such as location, contact number, and address |
| AdministrativeRecord_03 | View profiles of health professionals, such as name, education, and specialization |
| AdministrativeRecord_04 | Paying for medical expenses |
| AdministrativeRecord_05 | View information related to health insurance |
| MedicationsManagement_01 | View information related to past or current medicine, such as medicine names, dosages, and classifications |
| MedicationsManagement_02 | View information regarding prescriptions, such as the name of the medicine and the physician who wrote the prescription |
| MedicationsManagement_03 | Purchase medicines according to the prescription |
| MedicationsManagement_04 | Set a schedule and reminder of medicine consumption |
| Communication_01 | Send messages or chat with the physician |
| Communication_02 | Receive notifications via email/SMS/text when a message is replied to by a physician |
| Communication_03 | Send messages or chat with support groups and family members |
| ApointmentManagement_01 | Schedule appointments with the physician |
| ApointmentManagement_02 | View the history of appointments with the physician |
| ApointmentManagement_03 | Set a reminder or notification for an appointment |
| Education_01 | Access educational content or health tips |
| SelfHealthMonitoring_01 | Input personal health data such as weight, height, exercise, and food consumption |
| SelfHealthMonitoring_02 | Input vital signs data such as blood pressure, blood sugar, and heart rate |
| SelfHealthMonitoring_03 | Access health calculators, such as body mass index (BMI), calories, and disease risk |
| SelfHealthMonitoring_04 | View data visualization of personal health data |
| SelfHealthMonitoring_05 | Connecting wearable devices such as smartwatches or blood glucose meters |
| SupportingFunction_01 | Backup health information on the PHR application |
| SupportingFunction_02 | Access a guide that describes the features of the PHR application |
| SupportingFunction_03 | Option to show or hide certain health information according to users’ needs |
