# Supplementary material for: Integrated Personal Health Record in Indonesia: Design Science Research Study
Source: JMIR Med Inform. 2023 Mar 14;11:e44784. doi: 10.2196/44784 (PMC10131695; doi:10.2196/44784)
Supplement: Multimedia Appendix 6 [file medinform_v11i1e44784_app6.docx]

## **Multimedia Appendix 6. Summary of the PHR module and functionality based on the requirements of health organizations and patients**

| **Module** | **Functionality** | **Primary health facility** | **Hospital** | **Health regulator** | **Health application vendor** | **Patient** | **Total** |
| --- | --- | --- | --- | --- | --- | --- | --- |
| Health record | Results of physical examination or lab test | x | x |  |  | x | 3 |
|  | Medical history | x | x | x | x | x | 5 |
|  | Referral |  | x |  |  | x | 2 |
|  | Vaccination |  |  |  | x | x | 2 |
| Administrative record | Patient profile |  |  |  |  | x | 1 |
|  | Health facility profile |  | x | x | x | x | 4 |
|  | Physician profile |  |  |  |  | x | 1 |
|  | Health Insurance |  | x |  |  | x | 2 |
|  | Payment and billing | x | x |  | x | x | 4 |
| Medications management | Medication history | x | x |  |  | x | 3 |
|  | Medicine reminder | x |  |  |  | x | 2 |
|  | Medicine order | x | x |  | x | x | 4 |
| Communication | Messaging (text and/or video) | x | x | x |  | x | 4 |
| Appointment management | Registration | x | x |  | x | x | 4 |
|  | Queue | x | x | x |  |  | 3 |
|  | Appointment history |  |  |  |  | x | 1 |
|  | Reminder or notification |  |  |  |  | x | 1 |
|  | Ambulance |  |  |  |  | x | 1 |
| Education | Health article | x | x | x |  | x | 4 |
| Self-health monitoring | Health data tracking | x | x |  |  | x | 3 |
|  | Health dashboard |  |  |  |  | x | 1 |
|  | Health calculator |  |  |  |  | x | 1 |
|  | Early warning notification |  | x |  |  | x | 2 |
| Emergency | Emergency contact |  |  |  |  | x | 1 |
| Security | Authentication |  |  |  | x | x | 2 |
|  | Authorization |  |  |  | x | x | 2 |
|  | Audit log | x | x |  | x |  | 3 |
|  | Backup | x | x | x |  | x | 4 |
| Supporting function | User manual |  |  |  |  | x | 1 |
|  | Offline functionality |  |  | x |  |  | 1 |
