# Supplementary material for: Integrated Personal Health Record in Indonesia: Design Science Research Study
Source: JMIR Med Inform. 2023 Mar 14;11:e44784. doi: 10.2196/44784 (PMC10131695; doi:10.2196/44784)

## **Multimedia Appendix 7. Process flows in PHR**

1. *Health Care Process*


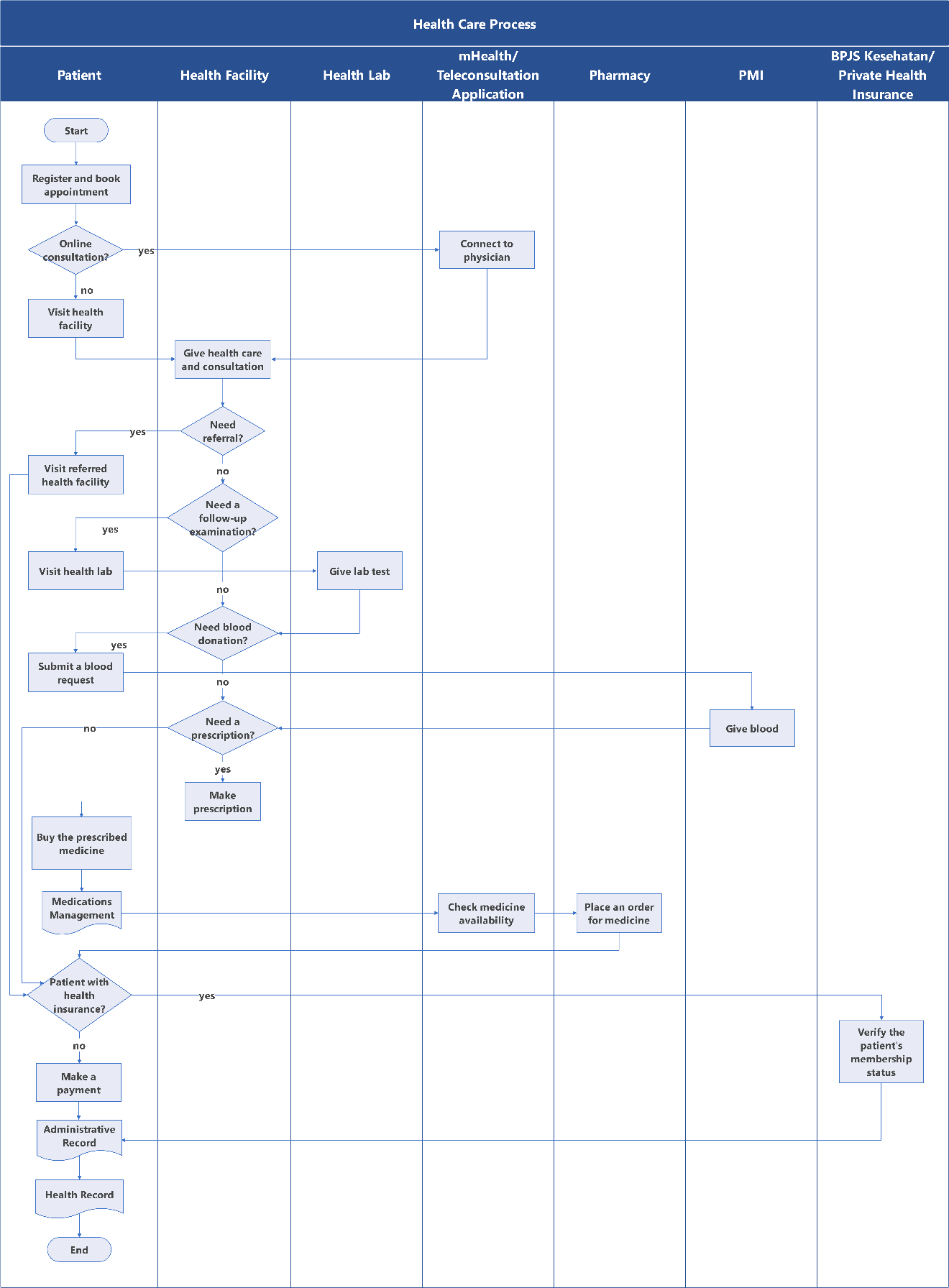


1. *Self-health Monitoring Process*


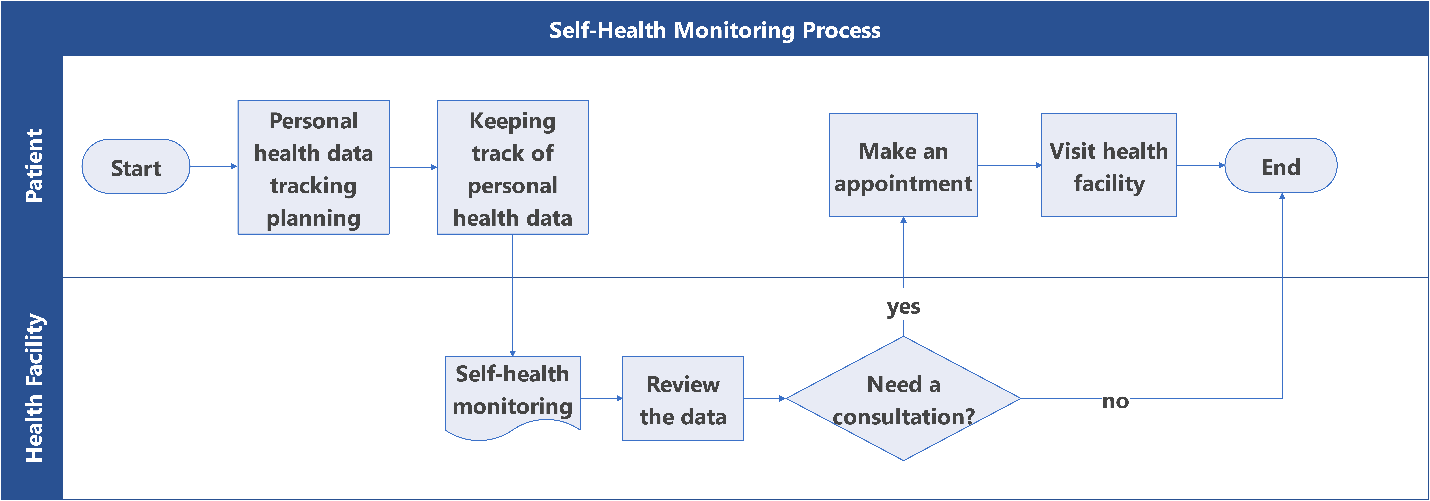


1. *Vaccination Process*


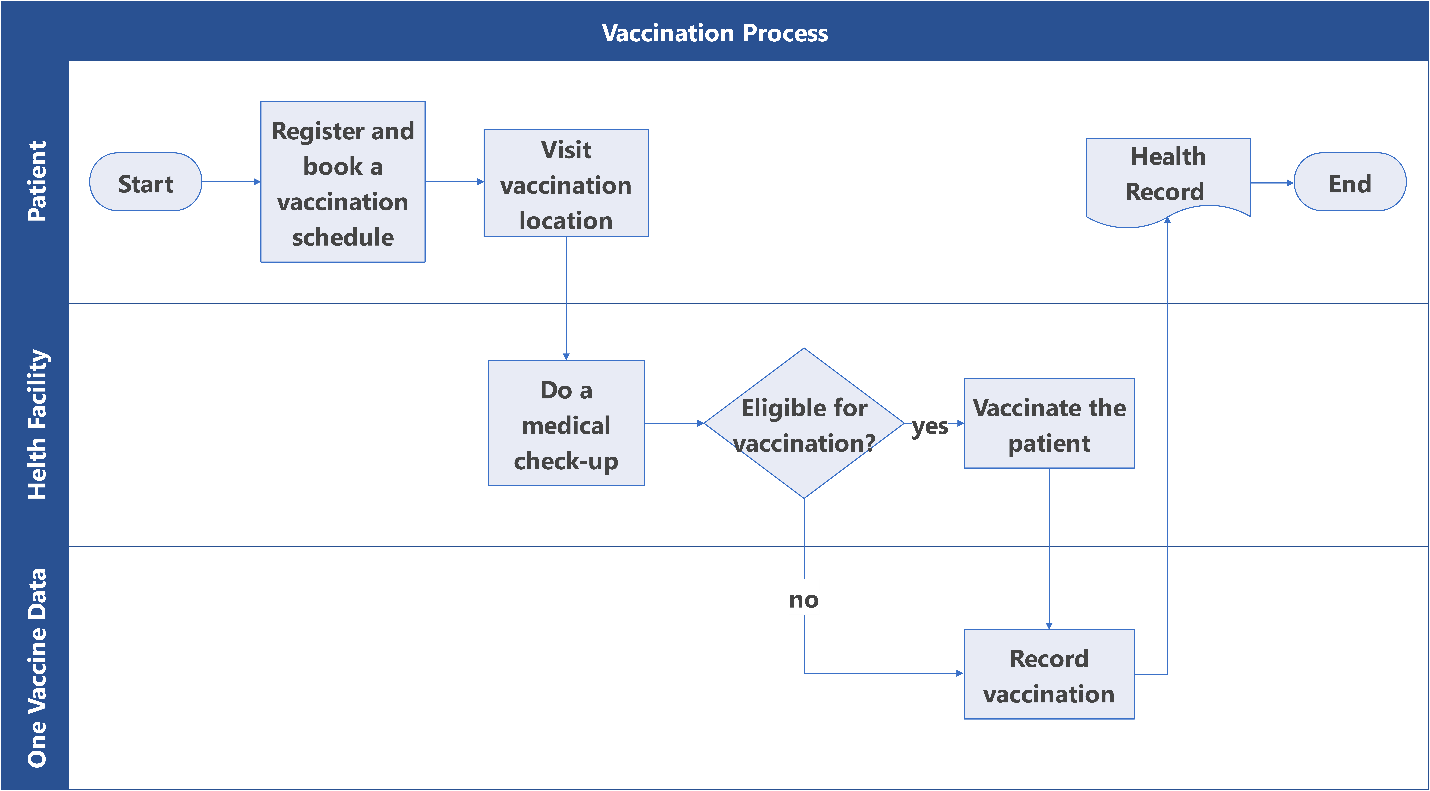


1. *Home Care Process*


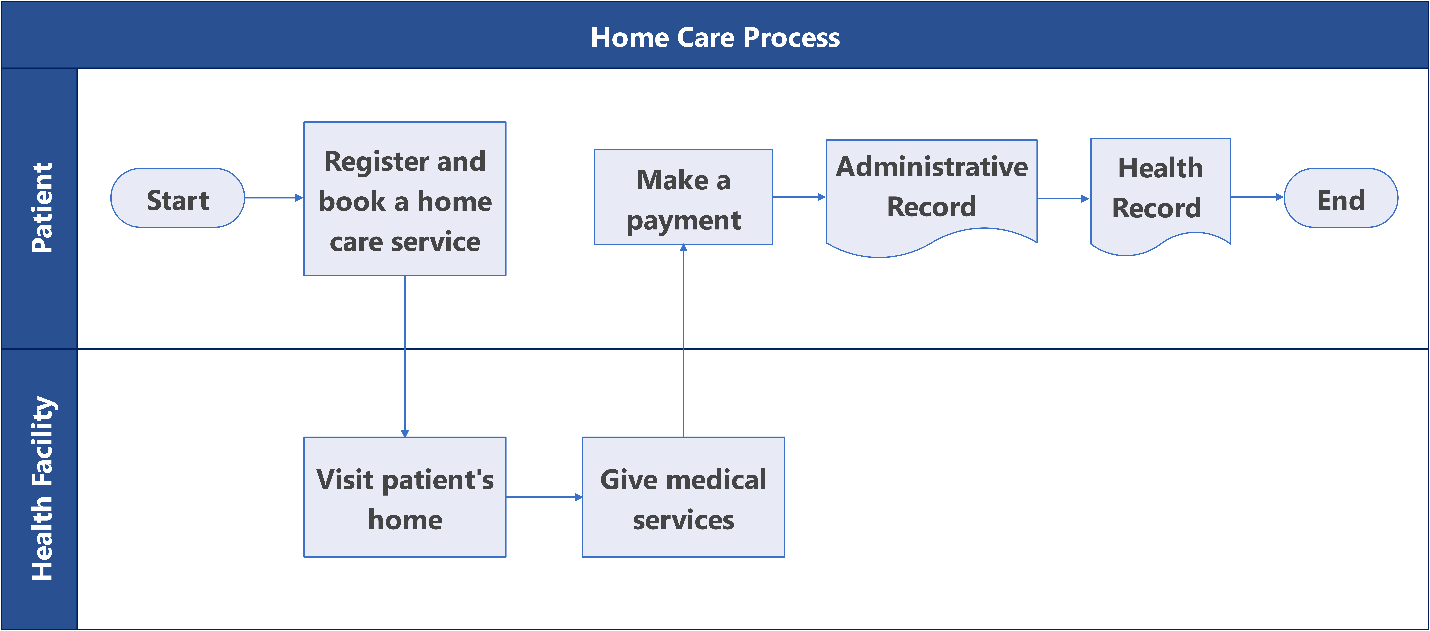

Supplement: Multimedia Appendix 7 [file medinform_v11i1e44784_app7.docx]
