# Supplementary material for: Integrated Personal Health Record in Indonesia: Design Science Research Study
Source: JMIR Med Inform. 2023 Mar 14;11:e44784. doi: 10.2196/44784 (PMC10131695; doi:10.2196/44784)
Supplement: Multimedia Appendix 8 [file medinform_v11i1e44784_app8.docx]

## **Multimedia Appendix 8. Module and functionality in PHR**

| **Module** | **Functionality** | **Description** | **Data Provider** | **Data Consumer** |
| --- | --- | --- | --- | --- |
| Health record | Medical resume | View the results of the medical examination and treatment as well as the patient’s diagnosis history | SIMRS, SIMPUS | PHR |
|  | Referral | View referral history | SISRUTE | PHR |
|  | Vaccination | View vaccination history | SIMRS, SIMPUS, Satu Data Vaksin | PHR |
|  | Homecare | Book health services at home | SIMRS, SIMPUS | PHR |
|  | Blood donors | Access information related to blood donation | PMI | PHR |
|  | Family planning | Access information related to family planning (KB) programs | SIMRS, SIMPUS | PHR |
| Administrative record | Patient profile | Manage patient demographic data such as name, gender, date of birth, and contact number | Dukcapil | PHR |
|  | Health facility profile | View health facility information, such as location, contact number, address, and health services provided | SIMRS, SIMPUS | PHR |
|  | Physician profile | View physician profiles such as name, education, and specialization | SIMRS, SIMPUS | PHR |
|  | Health insurance | View information on the national health insurance (JKN) for BPJS kesehatan patients or other health insurance | BPJS kesehatan, Private health insurance | PHR |
|  | Payment and billing | Make a payment of medical expenses and view the number of medical expenses | SIMRS, SIMPUS, Billing gateway | PHR |
| Medications management | Medicine history | View the history of medicine that have been or are being taken by the patient | SIMRS, SIMPUS | PHR |
|  | Medicine order | Order prescribed or generic medicines | mHealth/ Teleconsultation application | PHR |
|  | Medicine scheduler and reminder | Set schedules and reminders to take medicine | PHR | - |
| Communication | Messaging (text and/or video call) | Send messages to the physician via text or consultations by video call | mHealth/ Teleconsultation application | PHR |
| Appointment management | Registration and appointment | Schedule an appointment for a consultation with a physician in a health facility or book a schedule for a health lab | mHealth/ Teleconsultation application | PHR |
|  | Queue | View the order in which patients are treated after registering an appointment | mHealth/ Teleconsultation application | PHR |
|  | Appointment history | View the history of appointments | mHealth/ Teleconsultation application | PHR |
|  | Appointment reminder | Set reminders or notifications for upcoming appointments | mHealth/ Teleconsultation application | - |
|  | Ambulance | Nearest ambulance service contact | SPGDT | PHR |
| Education | Health article | Information on disease problems or health tips that can be tailored to the patient's condition and preferences | Ministry of Health | PHR |
| Self-health monitoring | Health data tracking | Input personal health data such as vital signs, physical activities, food consumption, and other health data according to patient needs | Wearable devices, PHR | Health facility |
|  | Health calculator | Health calculator to see health values such as body mass index (BMI), calories, and disease risk | PHR | - |
|  | Health screening | Conduct independent health screening and view health screening history | PIS-PK, PHR | PHR |
|  | Health dashboard | View personal health data visualization | SPGDT | PHR |
|  | Early warning notification | Displays warnings and potential health problems | PHR | - |
| Emergency | Emergency contact | Emergency contacts that can be reached by patients | SPGDT | PHR |
| Security | Authentication | User authentication process to gain access to PHR applications, such as passwords, biometrics, and face recognition | - | - |
|  | Authorization | Allows access to some of the information on PHR to other individuals or parties | - | - |
|  | Audit logs | See activity in the PHR app and who has access to certain data | - | - |
|  | Backup | Health information backup | - | - |
| Supporting function | User manual | A guide that explains the features of the PHR app | - | - |
|  | Offline functionality | Synchronize data online and offline environment | - | - |

**Notes:**

- SIMRS=*Sistem Informasi Manajemen Rumah Sakit* or Hospital Information System
- SIMPUS=*Sistem Informasi Puskesmas* or Primary Health Care Information System
- SISRUTE=*Sistem Informasi Rujukan Terintegrasi* or Integrated Referral Information System
- PMI=*Palang Merah* Indonesia or Indonesian Red Cross
- BPJS Kesehatan= *Badan Pelaksana Jaminan Sosial Kesehatan* or Social Security Agency for Health
- SPGDT= *Sistem Penanggulangan Gawat Darurat Terpadu* or Integrated Emergency Management System
- PIS-PK=*Program* Indonesia *Sehat dengan Pendekatan Keluarga* or Healthy Family Application
