# Supplementary material for: Integrated Personal Health Record in Indonesia: Design Science Research Study
Source: JMIR Med Inform. 2023 Mar 14;11:e44784. doi: 10.2196/44784 (PMC10131695; doi:10.2196/44784)
Supplement: Multimedia Appendix 9 [file medinform_v11i1e44784_app9.docx]

## **Multimedia Appendix 9. Data categories with data groups and descriptions in the PHR**

| **Data Category** | **Data Group** | **Description** |
| --- | --- | --- |
| Master | Patient | Patient’s demographic data. |
| Master | Health facility | Health facility data. |
| Master | Physician | The physician who works in health facilities or other health service providers. |
| Master | Polyclinic | Type of health service or polyclinic owned by the health facility. |
| Master | Health insurance | BPJS kesehatan or other private health insurance. |
| Master | Health article | Health article data. |
| Master | Medicine | Medicine data that was given by the physician to the patient. |
| Transaction | Medical record | A summary of the patient’s medical examination at the health facility. |
| Transaction | Vaccination | Vaccinations that have been done by the patient. |
| Transaction | Referral | Patient referral data from a health facility to another health facility. |
| Transaction | Homecare | Homecare service data. |
| Transaction | Blood donors | Blood donation activities that have been carried out by the patient. |
| Transaction | Family planning | Family planning data. |
| Transaction | Medicine history | Past or current medicine consumption. |
| Transaction | Medicine order | Patient’s medicine order data. |
| Transaction | Medicine reminder | Notification of patient medicine consumption. |
| Transaction | Communication | Communication between the patient and the physician. |
| Transaction | Patient schedule | Data on patient consultation schedules with physicians or appointments with other health care providers. |
| Transaction | Health service schedule | Data on the availability of health services that can be booked by patients. |
| Transaction | Ambulance | Data on the availability of the nearest ambulance from the patient's location. |
| Transaction | Health data tracking | Personal health measurement or monitoring data inputted by the patient or connected to a wearable device. |
| Transaction | Health screening | Patient’s health screening data. |
| Reference | Diagnosis name | Diagnostic name reference data. |
| Reference | Blood type | Blood type reference data. |
| Reference | Physician specialization | Reference data for the type of physician specialization or expertise. |
| Reference | Type of health facility | Reference data for types of health organizations. |
| Reference | Gender | Gender reference data. |
| Reference | Type of health service | Reference data for types of health services. |
| Reference | Education | Reference data for the education level of health workers. |
| Reference | Ward | Village reference data. |
| Reference | Subdistrict | District reference data. |
| Reference | City | City reference data. |
| Reference | Province | Provincial reference data. |
